# Eastern African expressions of suffering: A new Cultural Scripts of Trauma Inventory

**DOI:** 10.1371/journal.pone.0358482

**Published:** 2026-09-25

**Authors:** Celestin Mutuyimana, Andreas Maercker

**Affiliations:** 1 Department of Psychology, Division of Psychopathology and Clinical Intervention University of Zurich, Zurich, Switzerland; 2 The Collegium Helveticum, Zurich, Switzerland; Universitat der Bundeswehr München: Universitat der Bundeswehr Munchen, GERMANY

## Abstract

**Introduction:**

Many people who have experienced trauma feel that PTSD criteria do not describe their experiences. They report culturally specific symptoms, in our new theory referred to as Cultural Scripts of Trauma, yet no study has systematically mapped these experiences or developed tools to measure them.

**Objectives:**

This study aims to map cultural scripts of trauma suffering and to develop and validate the Cultural Scripts of Trauma Inventory (CSTI) across Eastern African countries, including Rwanda, Uganda, Tanzania, and Kenya.

**Methods:**

A qualitative study involving 115 participants across nine focus groups of trauma survivors and experts was conducted to map the main cultural scripts of trauma experiences and generate initial pool of items. This was followed by a quantitative validation study involving 1,208 participants from Rwanda, Uganda, Tanzania, and Kenya. In an online survey, participants completed the pre-CSTI and additional measures for convergent and divergent validity, such as the International Trauma Questionnaire, Clinical Aspects of Historical Trauma, Purpose in Life Questionnaire, Subjective Vitality, and General Self-Efficacy.

**Results:**

The results yield 71 Cultural Scripts of Trauma Sufferings (CSTs). The confirmatory factor analysis confirms 15 most relevant CSTs regrouped in five factors such as cognitive, body related, frame of mind, meaning disruption and family stress demonstrating excellent model fit. Those constitute 15 item-inventory that showed strong overall reliability and validity. Additionally, the model exhibited configural, metric, and partial scalar invariance, confirming its consistency across various groups.

**Conclusion:**

This study is advancing culturally informed diagnosis and treatment of post traumatic sequelae by identification of culturally specific post trauma suffering and development and validation of CSTI in East African countries. It provides clinicians and researchers with a resource to adopt a model of beyond and including PTSD by offering a deeper understanding of post-traumatic stress in diverse cultural contexts.

## Introduction

Globally, over 70% of people are exposed to a traumatic event; however, only about 3.9% meet the criteria for Post-Traumatic Stress Disorder (PTSD) [1,2]. In Sub Saharan Africa more than 80% of the population have faced a series of cumulative, collective, and repetitive traumatic experiences [3,4]. As result around 15% are suffering from PTSD and Complex PTSD (CPTSD) [5,6]. However, help-seeking behavior among trauma survivors remains low despite the high prevalence of trauma related disorders [7,8]. A crucial issue is that survivors, in this context, often fail to recognize their experiences within the established symptom pattern of PTSD [9,10]. For example survivors in East Africa suffered from feeling of unspeakable sufferings, body pains, mistrust, bitterness and heart wounds [11]. Survivors in Namibia perceived their suffering as something standing inside them [12].These forms of suffering was conceptualized in a new theoretical framework as Cultural Scripts of Trauma (CST) [10,13–15].

CST is defined as the set of culturally specific post-traumatic symptoms that are systematically sequenced and causally interconnected [16]. These symptoms are shaped and intensified by cultural values, norms, and beliefs [9]. CSTs also vary across cultures, with certain symptoms salient in one country but not in another [14]. For example, research has found that among East African populations, mistrust, body-related symptoms, and family stress are particularly prominent, whereas in Swiss samples, the urge to function and perform is more salient. Cultural values also differ: in Switzerland, symptoms are more often related to the urge to hide and endure suffering, while in East African contexts, they are more closely associated with the suppression of suffering, concerns about community reputation, and Christian beliefs [10,11,17]. CST complements standardized trauma diagnosis by capturing culture-specific symptoms often not included in ICD and DSM criteria.

The DSM introduced the Cultural Concepts of Distress (CCD) framework to improve recognition of culturally shaped expressions of psychological suffering, including cultural syndromes, idioms of distress, and explanatory models. Similarly, ICD-11 acknowledged the importance of cultural context in the experience and expression of trauma-related disorders, including PTSD and complex PTSD. However, these frameworks do not fully capture how trauma survivors experience, interpret, and express traumatic suffering within specific sociocultural contexts. CST differs from idioms of distress and CCD in several important ways. Idioms of distress generally refer to culturally specific expressions or ways of communicating suffering, such as local metaphors or commonly used phrases [18]. Therefore, idioms of distress are not actual disorders and not permanent but rather reflect the way a community interprets symptoms through semantic language [19,20].

For example, Baksbat in Cambodia has been linked to PTSD in some research and has been interpreted by many scholars as a disorder. However, a study involving people with lived experience in Cambodia has shown that Baksbat is a normal response in their context, understood as an expression of fear of various threats, particularly fear related to political expression, and it does not require medical treatment. The authors argue that Baksbat has been pathologized without sufficient justification [19]. Similarly, in Rwanda, the concept of *Ihahamuka*, although used for many years, was discontinued about a decade ago because it was deemed pejorative, as it was also used to describe cows with mental disturbances. The term Ihahamuka was replaced by *Ihungabana*, which referred to the displacement of certain elements in a person's psychological functioning. This term was later replaced by *Ibikomere*, meaning “heart wound” and *Ibikomere* is not a disorder but a way of expressing suffering (Mutuyimana & Maercker, 2020).

CCD, in turn, represent a broader category that includes not only idioms, but also cultural syndromes, explanatory models, and shared experiences recognized within a given cultural context [21]. In many studies, idioms of distress, cultural syndromes, and CCD are used interchangeably, which creates conceptual ambiguity and limits their practical usability [20,22]. This lack of clarity is further reflected in clinical practice, where limited interventions are developed specifically to address idioms of distress, as they are typically interpreted as surface-level expressions of underlying suffering rather than as clinically meaningful disorders.

Moreover, not all idioms of distress or CCD are inherently related to trauma; they may reflect a wide range of psychosocial difficulties. For example, *susto* in Latin America is often linked to spiritual disruption and has been described in three syndromic forms: one resembling depression, another somatic disorders, and a third related to PTSD [23]. As a result, trauma-specific symptoms that are culturally shaped may be overlooked or misinterpreted. This can lead to the underdiagnosis of culturally specific post-traumatic symptoms, as existing frameworks may fail to adequately capture how trauma manifests across different cultural contexts.

The CST approach addresses this gap. CST aims to complement universal diagnostic frameworks such as PTSD and CPTSD. While these models have demonstrated cross-cultural validity, they do not capture the full range of trauma-related experiences [14]. Based on that, CST highlights that trauma symptoms are not universally distributed or equally salient across cultures. In some contexts, culturally specific symptoms may have greater centrality and impact on individuals’ functioning than those emphasized in PTSD or CPTSD criteria. By advancing CST, this approach contributes a new layer to trauma theory: it moves beyond universal symptom lists toward understanding trauma as a culturally patterned, dynamic system of interconnected experiences. This has important implications for diagnosis, as well as for developing more culturally responsive and effective interventions.

Unlike the identified salient symptoms of PTSD and CPTSD, which emphasize a limited set of trauma sequelae derived largely from Western contexts [24] the cultural scripts of trauma sufferings and their corresponding measurements have yet to be systematically established. The absence of psychological measures for cultural scripts of trauma sufferings leads to an underestimation of the prevalence of posttraumatic disorders in highly traumatized populations. A cross cultural research have highlighted a paradox in the global distribution of trauma-related mental health issues, where individuals exposed to higher levels of traumatic events often exhibit fewer PTSD symptoms compared to those with less exposure [25]. This paradox does not imply that these individuals are not suffering, but rather that they may be experiencing other culturally specific symptoms that require special attention and recognition [26]. This gap carries significant implications for the lives of trauma survivors, as well as for their access to and utilization of mental health services.

The aim of this study is to map the cultural scripts of trauma experiences and to develop and validate the Cultural Scripts of Trauma Inventory (CSTI) across Eastern African countries, including Rwanda, Uganda, Tanzania, and Kenya. This study will assist researchers and clinicians in identifying not only standardized symptoms of PTSD but also culturally specific manifestations of trauma. By prioritizing the voices of individuals with lived experiences, the study ensures that their full experiences are captured. Once recognized by mental health practitioners, trauma survivors are more likely to trust and engage with mental health services. As a result, this approach has the potential to reduce the prevalence of post-traumatic sequelae and improve overall mental health outcomes.

## Materials and methods

### Qualitative study

#### Interview protocol guide.

The interview guide was developed basing on existing literature on trauma and cultural expressions of distress, as described in Cultural Script of Trauma (CST) (See S1 File). It included open-ended questions exploring the types of traumatic experiences, the symptoms or changes following these events, and how participants understood and related these symptoms to their cultural values and beliefs. For mental health practitioners and spiritual or traditional healers, the same questions were used, focusing on how they observe these experiences and symptoms in their clients.

#### Data collection procedure.

The study recruited adult trauma survivors and trauma experts from four countries: Rwanda, Uganda, Tanzania, and Kenya. The inclusion criteria for trauma survivors required them to experience trauma-related symptoms, regardless of whether they were receiving psychotherapeutic treatment, and to perceive themselves as fully connected to their cultural values, norms, and beliefs. Participants experiencing a mental health crisis or with psychotic disorders were excluded. Trauma survivors were recruited with the assistance of local NGOs working with trauma survivors or through psychotherapists. Trauma experts included psychotherapists, medical doctors, psychiatric nurses, and social workers who had experience of supporting trauma survivors. Additionally, traditional and spiritual healers who had provided care to trauma patients were also recruited. Participants were brought together in focus group discussions depending on their group: experts or trauma survivors. In Rwanda and Kenya, the discussions were conducted in person, while in the other countries, they were held in person or online via Zoom. Trauma survivors were asked to describe their post-traumatic stress experiences, whereas trauma experts discussed the experiences of their patients. Participants were also asked to explain how their sufferings or the sufferings of their patients are connected to the cultural values, norms, and beliefs in their communities. Each focus group session lasted between 2 and 2.5 hours and was audio recorded for analysis and or written in case of trust issue for recording. The interviews were conducted by the first author together with a local psychologist in each country. They were carried out in the participants’ preferred languages: Kinyarwanda in Rwanda; English in Uganda; and both English and Swahili in Kenya and Tanzania.

#### Qualitative data analysis.

Data were analyzed using thematic analysis frameworks [27] using MAXQDA. First, all transcribed and written data were cross-checked by two project members to ensure accuracy. Next, the qualitative material was independently coded by four coders focusing on recurring culturally specific trauma expressions and their meanings. The guiding questions for coders were: What post-traumatic expressions or changes are reported by participants, and which dimensions of cultural trauma scripts do they belong to? What item can be derived from those changes/symptoms mentioned? (See S2 File). Interviews conducted in Kinyarwanda and Swahili were coded in the original languages by local coders fluent in those languages to preserve cultural and linguistic nuances, and the resulting codes were later translated into English. Interviews conducted in English were independently coded by two researchers and reviewed by a local coder to ensure contextual accuracy. Following the initial coding process, the first author reviewed all codes in collaboration with a local psychologist. Any discrepancies were discussed until consensus was reached, resulting in a finalized and culturally validated items

#### Item content validity assessment.

The results yield initial pool of 98 items after removing redundant, irrelevant, and overlapping content, and evaluating items for clarity, and cultural relevance across sites. A pilot testing phase with 5 people in each country assessed item comprehensibility and cultural relevance. Their results indicated that all items were deemed resonant. The items in Kinyarwanda and Swahili were translated into English using forward and backward translation procedures. The final English version, along with the corresponding original-language items, was sent to eight experts from Rwanda, Kenya, Tanzania, and Uganda. This expert review led to the removal of 27 items (10 redundant, 12 unclear, and 5 with a Content Validity Index below 0.60). The remaining 71 items demonstrated strong content validity, with CVIs ranging between 0.80 and 0.90.

### Quantitative study

The first step was the translation of the tools. To ensure cultural appropriateness and comparability across settings, a rigorous translation process was used, including forward translation into the target languages by bilingual experts and backward translation into English by independent translators. Discrepancies were resolved through consensus with the research team in each country, and items were culturally adapted where necessary to preserve conceptual equivalence while improving clarity and relevance. The next step was the recruitment of participants through online surveys via social media like Facebook, WhatsApp, X (former Twitter), Instagram and email. Social media was used as a recruitment tool to reach a broader and more diverse sample of participants across different regions. To further enhance diversity and include individuals with limited access to online platforms, additional in-person recruitment was conducted with the assistance of local psychologists. The participants used a google form link to fill out the online questionnaire. Surveys were in Swahili in Tanzania, Kinyarwanda in Rwanda, English in Uganda, and Swahili and English in Kenya. Participants were required to provide informed consent by signing either an online or paper consent form prior to completing the 30–45-minute survey.

### Measures

#### Cultural scripts of Trauma Inventory (CSTI).

The resulting 15-item CSTI (subject to this paper) is scored on a 5-point Likert scale, ranging from 0 (never) to 4 (always). The measure is divided into five subscales: cognitive, frame of mind, body related, meaning disruption and family stress, with each subscale consisting of three items. The total score can range from 0 to 60, while subscale scores vary from 0 to 12. A score of 0 indicates no cultural related post traumatic symptoms, whereas higher scores indicate more significant cultural related post traumatic symptoms. In clinical settings, the significance of the score is linked to the severity of symptoms and corroborated with other clinical evidence.

#### PTSD and CPTSD.

The International Trauma Questionnaire [28] is an 18-items measure of PTSD symptoms based on criteria in the ICD-11 (WHO, 2019). The measure comprises two subscales: The first is used to assess core symptoms of PTSD (nine items), and the second relates to symptoms of CPTSD (nine items). Responses are rated on a 5-point Likert scale ranging from 0 (not at all) to 4 (extremely). The total score can range from 0 to 72, whereas subscale scores range from 0 to 36. In the present study, the reliability of the questionnaire for PTSD was excellent, Cronbach's α = .88 while for CPTSD was  .90.

### Historical trauma

The 20-item of Clinical Aspects of Historical Trauma Questionnaire [29] assesses cultural patterns of man-made collective trauma prevalent in Eastern Africa. Its items are scored on a 5-point Likert scale ranging from 0 (never) to 4 (always). The measure has five subscales: distrust, exhaustion, attributed damage, cultural loss, and reproaches, with each subscale consisting of three items. Total scores can range from 0 to 80, and subscale scores range from 0 to 16. The score of 0 indicates no clinical aspects of historical trauma, whereas higher scores indicate more significant clinical aspects of historical trauma, with cut-off point of 31 score. In the present study, the reliability of the questionnaire was excellent, Cronbach's α = .90.

### Purpose in life

To assess purpose of life the Existential Scale of the Purpose in Life Questionnaire (EPIL) [30] was used. It is a 7-point Likert scale ranging from 1 (the lowest level of a given response) to 7 (The highest level of the response). The high score indicates that the assessed person has a purposeful life. In the present study, the reliability of the questionnaire was excellent, Cronbach's α = .84.

### General self efficacy

To assess the ability of coping with the daily life stressors and a general set of expectations that the individual carries into new situations, nine items with higher factor loadings of general self-efficacy questionnaire [31] was used. It is a 5-point Likert scale ranging from 1 (Strongly disagree) to 5 (Strongly agree). The high score indicates that the assessed person has a higher general self-efficacy. In the present study, the reliability of the questionnaire was good, Cronbach's α = .78.

### Subjective vitality

To assess the culturally relevant concept of subjective vitality of the participants, a vitality questionnaire [32] was used. It is rated on a 7-point Likert scale with one item reverse scored. Higher scores indicate greater vitality. In this study, the scale showed good reliability (Cronbach’s α = .80).

### Social demographics

Variables of a newly developed brief demographic questionnaire are age, gender, education, marital status, employment category, country of residency, religion and type of traumatic exposure.

### Data analysis

Quantitative data [33] were analyzed using IBM SPSS and Amos (Version 29). Assumptions were checked, with no missing data, outliers, or normality issues. Descriptive statistics summarize sociodemographic characteristics. Though theory-based, an Exploratory Factor Analysis (EFA) using principal axis factoring with direct oblimin rotation was conducted. Parallel analysis and scree plot guided factor retention. Three items per factor with loadings > .50 and strong content relevance were selected. This approach follows guidelines to prioritize high-loading, meaningful items while keeping questionnaires brief [34]. Factors were validated via confirmatory factor analysis (CFA) with maximum likelihood estimation on two subsamples to check model fit and prevent overfitting. For EFA and CFA, the total sample was randomly split into two independent subsamples of 604 participants each. One subsample was used for EFA, and the second subsample was used for CFA. This split-sample (50%/50%) approach is commonly recommended in scale development when the sample size is sufficiently large, as it allows cross-validation of the factor structure while reducing the risk of overfitting. CFA was conducted only on the second subsample to confirm the factor structure identified in the EFA [35]. Internal consistency was assessed using Cronbach's alpha. Split-half reliability tested item consistency, and item analysis examined discrimination and difficulty. Construct and discriminant validity were evaluated using AVE [36] and HTMT [37]. Convergent and discriminant validity were tested using Pearson correlations. Measurement invariance was conducted to assess the questionnaire's validity and reliability across four countries. Following Byrne [38], CFA was first run in each country to ensure model fit. The model fit well in three countries, and results excluding the poorly fitting country aligned closely. Thus, measurement invariance analysis proceeded across all countries [38]

### Ethical considerations

This research was approved by Ethics Committee of the University of Rwanda, College of Medicine and Health Sciences, approval nr. 396/CMHSIRB/2023, Ethics Committee of the Daystar University Kenya, approval nr. DU-ISERC/09/05/2023/00086, ETH Zurich ethics committee Nr. EK 2023-N-308, University of Dar es Salaam ethical Committee Ref. No: AB3/12(B) and Makerere University School of Health Sciences ethical committee MAKSHSREC-2026–1903. The study conforms to the principles embodied in the Declaration of Helsinki.

### Inclusivity in global research

Additional information regarding the ethical, cultural, and scientific considerations specific to inclusivity in global research is included in the Supporting Information (S3 File).

## Results

### Qualitative results

#### Trauma experts’ characteristics.

The study included a total of 36 participants from Kenya (*n* = 10), Rwanda (*n* = 16), Tanzania (*n* = 5), and Uganda (*n* = 5). The sample comprised 21 females (58.3%) and 15 males (41.7%), representing a range of mental health and psychosocial professions, including clinical psychologists (*n* = 7), psychotherapists (*n* = 16), medical doctors (*n* = 3), social workers (*n* = 3), traditional healers (*n* = 3), and spiritual healers (*n* = 4). Participants’ ages ranged from 22 to 58 years (M = 40.05, SD = 9.98). Years of professional experience ranged from 1 to 20 years (M = 8.36, SD = 5.44).

#### Trauma survivors’ characteristics.

The sample consisted of 79 participants (age range = 21–83 years, M = 45.60, SD = 12.80) from Rwanda (*n* = 59), Uganda (*n* = 5), Kenya (*n* = 10), and Tanzania (*n* = 5). Participants included survivors of genocide (*n* = 20), war (*n* = 15), imprisonment (*n* = 13), ex-combatants (*n* = 8), accidents (*n* = 7), childhood trauma (*n* = 10), and conflict-related experiences (*n* = 5). Most participants were married (*n* = 50), followed by single (*n* = 18) and widowed individuals (*n* = 11). In terms of education, participants had primary education (*n* = 27), secondary education (*n* = 23), university-level education (*n* = 21), or no formal education (*n* = 8).

#### List of mapped cultural scripts of trauma suffering: Initial pool of items.

The following section presents the results that guided the item selection and formulation for the East African CSTI. The coded text segments were assigned to the following main categories: cognitions and affects, worldviews, interpersonal relationship problems, body-related symptoms, and meaning disruption and changes in family stress (see S4 File). Body-related symptoms such as headaches, back pain, dizziness, and tingling sensations were the most frequently reported, with nearly all participants. Both survivors and experts provided similar explanations for these complaints.

They described bodily symptoms as signs of silent suffering pain endured without sharing, healing, or finding ways to express distress. These accounts highlight the survivors’ need for a confidential, non-stigmatizing space in which to share their experiences.

Interpersonal relationship problems were the second largest category with twelve items. It includes difficulties with enemies (perpetrators), loss of trust in others, heightened competitiveness, and feelings of insufficient family and community support. For example, participants expressed sentiments such as*: “I cannot trust anyone,” “I feel that no one wants to support me,” and “I am glad when my enemy sees my successes.”* These statements illustrate the enduring impact of man-made trauma on interpersonal relationships and highlight the long-term scars it leaves on the social fabric.

The third category concerned changes in family interest and management. Participants described how traumatic experiences extend beyond the individual level to affect the entire family. Examples of such statements included: *“I feel that I have an empty family,” “I am aggressive towards close others,”* and *“I don’t know how to manage family resources.”* These accounts illustrate how trauma can deeply disrupt family functioning and contribute to a loss of hope within the family unit.

The fourth category, comprising ten items, was cognitions and affects. Although the symptoms in this category were primarily cognitive, they were often perceived in an interdependent manner. For example, participants stated: *“I have no more love,” “I feel I can’t apologize”* and *“I feel resentment or revenge.”* These expressions highlight negative cognitions directed toward others. At the same time, participants also reported personal negative cognitions, such as *“I feel that I am cruel”* and *“I feel that I have lost my dignity.”* This suggests that in collectivistic communities, negative cognitions are not only self-oriented but also relationally oriented toward others.

The fifth category concerned changes in worldview. Participants from the East African sample reported uncertainty about the future, perceptions of human unfairness, and deep disappointment with both the world and God. For example, some expressed: *“I believe there is no need to plan for tomorrow” and “I doubt that there is a God.”* These statements reflect how trauma disrupts an individual’s system of beliefs. In the East African context, this system is often a triple belief structure encompassing the self, the community, and God. Supporting trauma survivors therefore requires interventions that help to restore and rebuild this interconnected belief system.

Interestingly, trauma survivors also described negative experiences related to meaning and purpose in life. For example, participants stated, *“I do not need to strive for myself” and “I give up easily,”* reflecting a diminished sense of agency, interconnectedness, and existential meaning following traumatic experiences. All these cultural scripts of suffering are closely linked to cultural values and beliefs such as enduring sadness in silence, preserving community reputation, and maintaining Christian faith.

### Quantitative results

#### Participant demographic characteristics.

This study included 1,208 participants from four East African countries. The mean age of the participants was 32.63 years (*SD* = 10.85), with a range from 18 to 81 years. Of the total sample, 34.1% (*n* = 412) were from Tanzania, 26.5% (*n* = 320) from Rwanda, 21.2% (*n* = 256) from Uganda, and 18.2% (*n* = 220) from Kenya. The sample consisted of 63.2% (*n* = 763) female participants, while 0.7% (*n* = 9) identified as a gender other than male or female. In terms of marital status, 52% (*n* = 628) were single, 37.3% (*n* = 451) were married and living with their partner, and 10.7% (*n* = 129) were divorced. Regarding education, 8.6% (*n* = 104) had completed elementary school, 19.4% (*n* = 234) had finished high school, 59.9% (*n* = 723) held a bachelor’s degree, 10.8% (*n* = 130) had a master’s degree, and 1.4% (*n* = 17) had a PhD.

Employment status revealed that 14.1% (*n* = 170) were unemployed, 31% (*n* = 375) were students, and 54.9% (*n* = 663) were employed. Regarding religious affiliation, 1.2% (*n* = 14) followed traditional religions, 2.3% (*n* = 28) had no religious affiliation, 16.9% (*n* = 204) were Muslim, and 79.6% (*n* = 962) were Christian. Most participants, 72.7% (n = 878), lived in urban areas.

#### Exploratory factor analysis of item pool.

**Fig 1:**
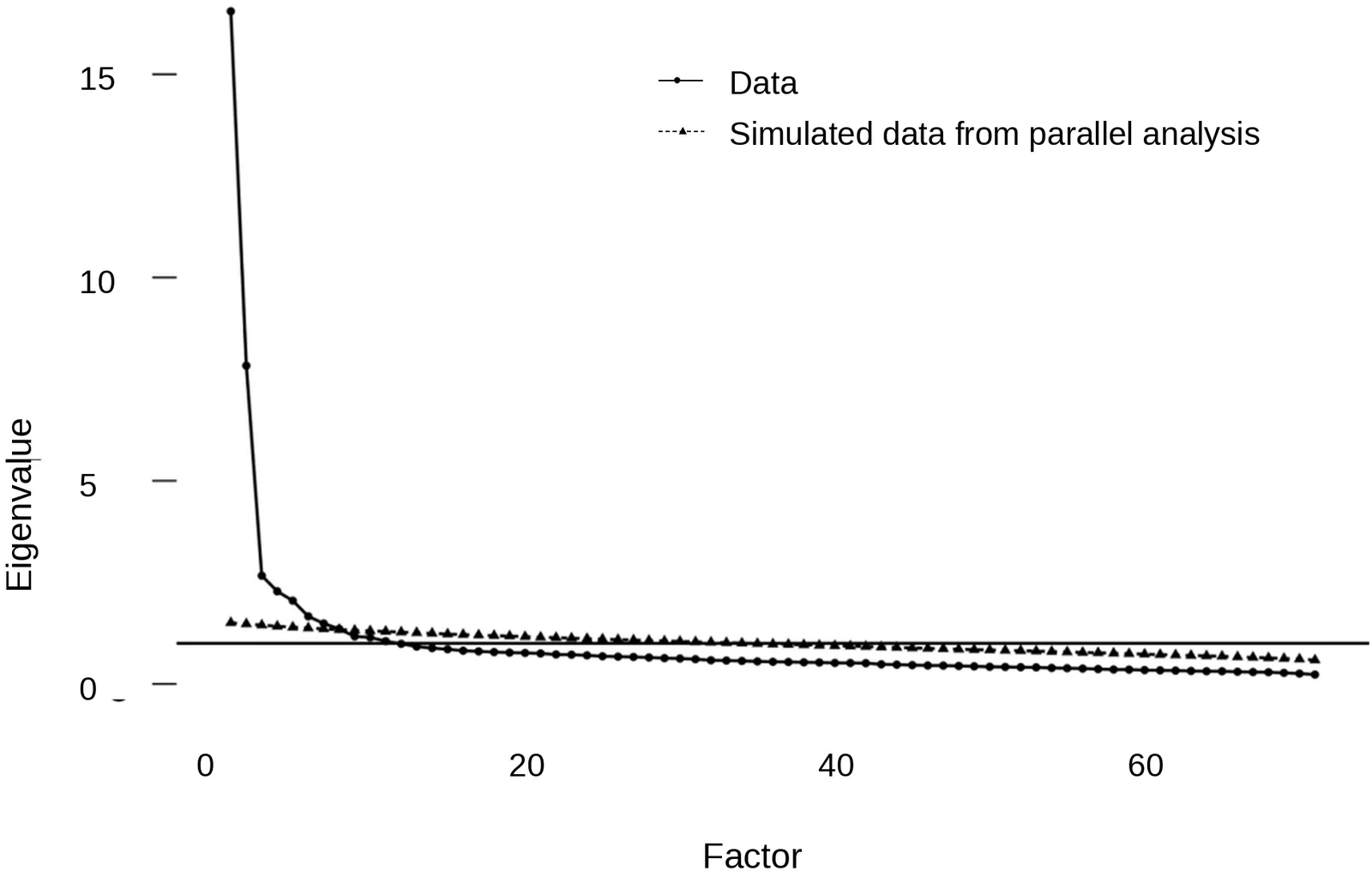
Scatter plot of parallel analysis.

An exploratory factor analysis (EFA) of the CSTI initially yielded a nine-factor solution with eigenvalues of 16.55, 7.83, 2.28, 2.10, 2.05, 1.66, 1.48, 1.35, and 1.17. To determine the optimal number of factors, we conducted a parallel analysis, which suggested up to eight factors exceeding randomly generated eigenvalues (1.50, 1.47, 1.44, 1.41, 1.39, 1.37, 1.35, 1.33, 1.32, 1.32). However, inspection of the scree plot (Fig 1), together with the parallel analysis scree line, indicated a clear break after the fifth factor, suggesting a more parsimonious five-factor solution.

In addition, the last three factors of the higher-order solutions explained less than 1% of variance each and showed unstable item patterns with substantial cross-loadings and conceptual overlap. Simulation results further indicated that eigenvalues beyond the fifth factor closely approximated those expected under random data, supporting retention of five factors.

Based on these converging criteria, a final EFA was conducted specifying a five-factor solution, which demonstrated a stable and interpretable structure consistent with the theoretical framework of the Cultural Script of Trauma (CST). This final five-factor solution was subsequently used for CFA and item selection. For the CFA, the three highest-loading and most conceptually representative items per factor were retained, resulting in a final 15-item CSTI (Table 1).

**Table 1:** Final 15-items Cultural Script of Trauma Inventory in all languages (CSTI) (S5 File).

No	English	Score				
		0	1	2	3	4
	**Cognitive**					
**1**	I have no more love					
**2**	I no longer feel like a person like others					
**3**	I feel that I have lost my dignity					
	**Frame of mind**					
**4**	I believe that there is no need to plan for tomorrow					
**5**	I feel that no one wants to support me					
**6**	I feel like I do not have a role model in my life					
	**Body related**					
**7**	I have sleeping problems					
**8**	I have headaches					
**9**	I feel tingling in my skin					
	**Meaning disruption**					
**10**	I do not need to strive for myself					
**11**	Since that time, I have not achieved any success.					
**12**	I do not have faith that everything is possible.					
	**Family stress**					
**13**	I feel that I have an empty family					
**14**	I am aggressive towards close others					
**15**	I don't know how to manage family resources					

Instructions: The following statements describe problems that people sometimes report in response to traumatic or stressful life events. Please read each statement carefully, then circle one of the numbers to the right to indicate how much you have been bothered by that problem in the past month. Write down one event that affected you more deeply than any other, and use it as a reference point when answering the questions: e.g., genocide, childhood trauma, war, accident, imprisonment etc.

**Notes:** Items 2, 4, 8, 10, 14 were found to be consistent in all countries as shown by partial scalar invariance.

Item Scoring: **0**: Strongly Disagree; 1: Disagree; 2: Neutral; 3: Agree; 4: Strongly agree

Total Scale Scoring*:* The CSTI ranges from 0 to 60, with 0 indicating absence of CST symptoms. A score ≥2 on any subscale, especially when accompanied by clinical symptoms or functional impairment, is considered clinically significant. In clinical settings, even one CST symptom scored ≥2 may warrant clinical attention.

#### Confirmatory factor analysis.

Table 2 presents the CSTI model fit indices. Model fit was considered adequate if it met the following criteria: χ²/df ≤ 3.0, RMSEA ≤ .08, SRMR ≤ .10, CFI, TLI, and GFI ≥ .95 (Kline, 2011). The five-factor model showed good fit for each factor and the overall structure. As expected, the single-factor model fit poorly, with all indices below cutoff values. Results were consistent across countries, except Kenya. However, CFA excluding Kenya produced similar outcomes, and reliability and construct validity remained strong, supporting the questionnaire's use in Kenya (see Table 2).

**Table 2:** CFA fit indices.

Fit index	Four-country model (5-factor)	One-factor model (4 countries)	Rwanda (5-factor)	Uganda (5-factor)	Tanzania (5-factor)	Kenya (5-factor)	Three-country model, excluding Kenya (5-factor)
χ²/df	2.18	15.10	1.35	1.56	1.62	1.58	2.18
CFI	0.98	0.77	0.99	0.97	0.94	0.96	0.97
TLI	0.97	0.73	0.98	0.96	0.93	0.95	0.97
GFI	0.98	0.91	0.98	0.96	0.96	0.99	0.98
RMSEA	0.035	0.10	0.024	0.042	0.050	0.039	0.035
SRMR	0.032	0.070	0.032	0.050	0.045	0.046	0.032

*Note.* The four-country model represents the proposed five-factor structure tested across Rwanda, Uganda, Tanzania, and Kenya. The one-factor model represents a single-factor alternative tested across all four countries. Country-specific models use the five-factor structure. The three-country model excludes Kenya.

#### Tests of reliability.

Table 3 shows the internal consistency of the final CSTI and its subscales. The total scale demonstrated strong reliability (Cronbach’s α = .85). Subscales showed adequate consistency: Cognitive (α = .76), Frame of Mind (α = .68), Body-Related Symptoms (α = .74), Difficulties in Meaning Disruption (α = .72), and Family Stress (α = .67). Split-half reliability was good, and item analysis indicated strong discrimination and difficulty indices. High factor loadings further confirmed item reliability (see Table 4).

**Table 3:** Item analysis and reliability.

Items	DI	Item α value	ID (Mean)	Items factor loadings
Item 1	0.57	0.85	1.6	0.80
Item 2	0.607	0.84	1.42	0.86
Item 3	0.625	0.84	1.43	0.91
Item 4	0.413	0.85	1.52	0.76
Item 5	0.506	0.85	1.62	0.71
Item 6	0.365	0.86	1.82	0.58
Item 7	0.511	0.85	1.74	0.86
Item 8	0.566	0.85	1.6	0.98
Item 9	0.565	0.85	1.45	0.74
Item 10	0.233	0.86	1.35	0.61
Item 11	0.422	0.85	1.24	0.85
Item 12	0.416	0.85	1.18	0.76
Item 13	0.548	0.85	1.52	0.76
Item 14	0.568	0.85	1.46	0.75
Item 15	0.509	0.85	1.47	0.61
General reliability		0.85		

DI: discrimination Index, ID: Index of difficulty.

**Table 4:** Descriminant validity.

	Cognitive	Frame of mind	Body related	Meaning disruption	Family stress
Cognitive					
World view	0.835				
Body related	0.725	0.763			
Meaning Disruption	0.285	0.309	0.177		
Family stress	0.707	0.866	0.701	0.218	

This number represents Heterotrait-monotrait ratio (HTMT) values.

#### Tests of validity.

Table 4 presents the HTMT ratios, confirming discriminant validity for all subscales with values below  .85. Table 5 shows construct validity results, with each subscale demonstrating AVE > .40 and composite reliability > .60 (Fornell & Larcker, 1981).

**Table 5:** Construct validity.

Construct	
Cognitive and affects	0.5
Frame of mind	0.4
Body related	0.5
Meaning disruption	0.4
Family stress	0.4

This number represent Average variance extracted (AVE) to each dimension

Table 6 displays correlations between CSTI and other measures. CSTI was positively correlated with PTSD, CPTSD, and CAHTQ (convergent validity), and negatively correlated with purpose in life, self-efficacy, and subjective vitality (discriminant validity).

**Table 6:** Convergent and divergent validity.

	CSTI	CAHTQ	PTSD	DSO	Purpose in Life	General Self Efficacy	Subjective vitality
CSTI							
CAHTQ	.503^**^						
PTSD	.511^**^	.658^**^					
DSO	.465^**^	.494^**^	.551^**^				
Purpose in Life	−.509^**^	−.366^**^	−.441^**^	−.373^**^			
General Self Efficacy	−.517^**^	−.418^**^	−.465^**^	−.401^**^	.482^**^		
Subjective vitality	−.497^**^	−.278^**^	−.385^**^	−.411^**^	.603^**^	.559^**^	
Personal Mastery	−.233^**^	−.252^**^	−.261^**^	−.347^**^	.219^**^	.360^**^	.242^**^

** Correlation is significant at the 0.01 level (2-tailed).

*Note: CSTI: Cultural Script of Trauma Inventory, CAHTQ: Clinical Aspects of Historical Trauma Questionnaire, PTSD: Post Traumatic Stress Disorders, DSO: Disturbances in Self Organization*.

### Measurement of invariance

Table 7 shows that four types of invariances were tested across four East African countries, confirming the CSTI’s reliability and validity. Adequate model fit was based on ΔCFI ≤ 0.01, ΔMc NCI ≤ 0.01, and Δγ ≤ 0.2 [39]. Configural and metric invariance were fully supported, partial scalar invariance was achieved with five items, but residual invariance did not hold (Table 7).

**Table 7:** Measurement of invariance.

Model	Model description	χ²	df	CFI	gamma hat	Mc NCI	Δχ²	Δdf	sig.	ΔCFI	ΔMc NCI	Δgamma hat
1	Configural invariance	535.12	320.00	0.95	0.98	0.91						
2	Metric	634.34	350.00	0.94	0.97	0.89	99.22	30.00	>.001	0.01	0.02	0.01
3	Partial scalar	735.93	360.00	0.93	0.96	0.85	101.58	10.00	>.001	0.01	0.03	0.01
4	Partial residuals invariance	1029.15	405.00	0.88	0.93	0.77	293.22	45.00	>.001	0.05	0.08	0.03

*Notes: χ²: Chi Square, df: Degrees of Freedom, Comparative fit index (CFI) Δ: Difference, Mc NCI: McDonald’s non-centrality index, Significance of P value.*

## Discussion

This study aims to map culturally scripts of trauma sufferings and to develop and validate the Cultural Scripts of Trauma Inventory (CSTI) across Eastern African countries, including Rwanda, Uganda, Tanzania, and Kenya to enable culturally informed diagnostics beyond PTSD and CPTSD (see S6 File). The qualitative study identified 71 items, which were organized into six categories: cognitions and affects, interpersonal relationship problems, changes in worldview, body-related symptoms, meaning disruption, and changes in family interest and management. In contrast to the Swiss sample, which revealed cultural scripts of trauma largely overlapping with CPTSD symptoms [17], the East African sample demonstrated symptoms that were more culturally specific. This highlights why trauma survivors in developing countries often do not fully fit the conventional PTSD criteria.

Cognitive and affective symptoms were often relationally oriented, highlighting how negative thoughts and emotions in collectivistic communities affected by man-made trauma extend beyond the self to others. Therefore, interventions should address both personal and relational dimensions. Changes in worldview reflected disruptions in the triple belief system encompassing the self, community, and God. This has important implications for diagnosis and treatment, as effective interventions should aim to restore and rebuild this interconnected belief system.

Nearly all trauma survivors reported body-related symptoms, indicating silence embedded suffering that many describe as unspeakable which require the safe and non-stigmatizing spaces [11]. This need is particularly important in this community, and their voices should be acknowledged and prioritized. Notably, survivors also reported problems in meaning and purpose in life, in the statement like I do not need to stive for my life, and my future is not needed. Such statements demonstrate lack of resilience and determination, and they may also suggest a diminished sense of interconnectedness and meaning in life. The qualitative findings also indicated that trauma extends beyond the individual to affect family and community levels, suggesting that interventions should adopt a multisystemic approach [14]. Although PTSD criteria mainly focus on individual symptoms, the effects of trauma appear to extend beyond the individual level in many communities.

In this study, symptoms such as family stress and aggressiveness illustrate how trauma in East African settings can deeply disrupt family functioning and contribute to a loss of hope within the family unit—manifestations that are not reflected in the DSM and ICD. Nevertheless, these results corroborate with research in Western sample which has shown that trauma can affect parenting practices, partner and interpersonal relationships, and children’s well-being [40,41]. The only simple difference is that such effects in Western contexts are often limited to nuclear family. However, in the East African context, forms of family disruption such as experiences of an “empty family and mistrust” may extend beyond the nuclear family to encompass the broader kinship network and community systems, reflecting the more relational and interconnected nature of family structure.

While qualitative studies found six categories the CFA only confirm five categories: cognitive and affects, body-related, meaning disruption, family stress and frame of mind. “*Frame of mind*” was chosen as a label for three items grouped together after EFA because they reflect a common mindset in the region marked by a lack of future planning due to ongoing uncertainties and doubts about reliable support. This mindset stems from repeated trauma, which undermines confidence in stability and social networks, highlighting the need to help East African trauma survivors rebuild purpose and restore social connections. The interpersonal category was not identified as a distinct factor, likely because family-related stress was more prevalent, causing item overlap. Similarly, items related to changes in worldview did not emerge as a separate factor, as many of these items were captured under the broader “frame of mind” dimension. This may reflect the tendency of trauma survivors to focus on fears and uncertainties about the future.

The factors with the highest loadings were body-related and cognitive symptoms, emphasizing their importance in post-traumatic stress treatment in East Africa. CFA and measurement invariance showed excellent fit across four countries, supporting the tool’s reliability. Although the CFA fit for Kenya was somewhat less satisfactory, however, the Comparative Fit Index (CFI) was relatively acceptable, and other psychometric properties, including factor loadings, internal consistency, and overall factor structure, were satisfactory. This suggests that the scale retains acceptable structural validity in this context, but further studies should explore the reason for such heterogeneity.

It is important to note that the symptoms of lack of future planning, bitterness, headaches, lack of agency, and aggressiveness towards close relationships emerged as significant concerns across all countries studied, as indicated by the findings of partial scalar invariance. These symptoms highlight the pervasive impact of trauma on individuals’ mental health and interpersonal dynamics. Understanding these central symptoms and signs is crucial for developing effective, culturally sensitive interventions that can help individuals in these contexts regain agency, purpose in life, vitality, rebuild their social connections and resilience [42,43].

The inventory shows strong internal consistency and reliable items. Each factor demonstrates adequate reliability, allowing use as standalone measures. Construct and discriminant validity are confirmed, along with convergent and divergent validity, indicating the CSTI is an effective tool for assessing culturally relevant post-traumatic symptoms in this sample. Few elements of CST categories of cognitive and affective changes and interpersonal relationships not included in the inventory may overlap with core DSM symptom clusters for trauma-related disorders, although their expression differs. For example, hyperarousal may be reflected in experiences of nervousness and hypersensitivity, while negative alterations in cognition and mood are expressed as sadness. However, several CST features in same categories, such as revengeful thoughts and loss of pride, are not captured within standard DSM symptom categories. At the same time, several CST categories extend beyond DSM symptom organization, particularly frame of mind changes, meaning disruption, family-level stress, and body related symptoms. These domains reflect broader socio-cultural and existential dimensions of trauma that are not fully captured by DSM and ICD categories, which are primarily focused on individual psychopathology.

Overall, these findings support a dual perspective on trauma symptoms. First, some symptoms appear relatively universal across cultures, while their expression, salience, and meanings are culturally shaped and embedded within broader social, familial, and moral systems. Second, certain symptoms may be culturally specific and occur only within cultural contexts.

This study has several limitations. First, its retrospective design may introduce memory bias. As a self-report tool, the questionnaire may overlook some important symptoms. Although several countries were involved in its development, validation was limited to four. Further validation in other countries is needed. While the inventory identifies CST in East Africa, future research should examine the sequence and network of symptoms, as well as the diversity of symptom patterns across subcultural groups. Many participants are relatively young, particularly in the quantitative component, which may limit age diversity. However, this reflects regional demographics, where approximately 85–91% of the population is below 45 years. To further mitigate this limitation, the qualitative sample is more age diverse. Recruitment varied across countries using both online and in-person methods, which may introduce some inconsistency, but also enabled broader reach across different contexts. Although some participants were students based in urban areas due to the location of universities, most maintain strong rural ties or lived rural experience, reducing potential urban bias and supporting contextual relevance. Furthermore, the ≥ 2 threshold mentioned was used as a pragmatic indicator of symptom presence rather than a diagnostic cutoff. Clinically meaningful cutoffs require validation in clinical samples using sensitivity and specificity analyses and remain a direction for future research.

## Conclusions and implications

Culture plays a crucial role in the treatment of patients experiencing post-traumatic symptoms. While PTSD and CPTSD are the most recognized diagnostic categories globally, with established and validated measurement tools, there is a pressing need to consider culturally specific experiences in the assessment and treatment of trauma. This study introduces further culturally specific post traumatic sufferings and a new measurement for assessing culturally relevant post-traumatic experiences in three languages, which encourages clinicians and researchers to move beyond a purely universal model of mental health diagnosis and treatment. The 15-item CSTI should be used in addition to the existing 18-item ITQ for ICD-11 PTSD and CPTSD. Future validation of this measurement in other African countries and corresponding studies in other cultural regions in the world is vital for broadening its clinical use.

## Supporting information

S1 File Interview Guide. (XLSX)

S2 File CST code book final. (XLSX)

S3 File Inclusivity in global research. (DOCX)

S4 File List of mapped cultural scripts of trauma suffering: initial pool of items. (DOCX)

S5 File Cultural Script of Trauma Inventory (CSTI). (DOCX)

S6 File Complete description of qualitative dataset. (DOCX)
