# Supplementary material for: Eastern African expressions of suffering: A new Cultural Scripts of Trauma Inventory
Source: PLoS One. 2026 Sep 25;21(9):e0358482. doi: 10.1371/journal.pone.0358482 (PMC13614634; doi:10.1371/journal.pone.0358482)
Supplement: Supplementary file 1 [file pone.0358482.s003.docx]

Inclusivity in global research

PLOS’ policy on inclusivity in global research aims to improve transparency in the reporting of research performed outside of researchers’ own country or community and ensures that PLOS publications reporting global research adhere to high standards for research ethics and authorship. Authors of relevant research articles may be asked to complete the questionnaire below, which outlines ethical, cultural, and scientific considerations specific to inclusivity in global research. This questionnaire may be requested when researchers have travelled to a different country to conduct research, if research uses samples collected in another country, research with Indigenous populations or their lands, or if research is on cultural artefacts. Researchers travelling to another country solely to use laboratory equipment will not normally be required to complete the questionnaire. However, the questionnaire can be requested at the journal’s discretion for any submission – if you have been requested to complete this questionnaire by the PLOS journal you submitted to, please do so.

Please complete the questionnaire below and include this as a Supporting Information file with your manuscript. Note that if your paper is accepted for publication, this checklist will be published with your article in the supporting information files. Please ensure that you reference the checklist in the main body of your manuscript. We suggest adding a subsection ‘Inclusivity in global research’ to your Methods section and adding the following sentence: “Additional information regarding the ethical, cultural, and scientific considerations specific to inclusivity in global research is included in the Supporting Information (SX Checklist)”

The questions have been designed to be applicable to a wide range of study types, and there are subsections for both human subjects research and non-human subjects research. If any of the questions are not relevant to your research please mark them as “N/A” as appropriate.

**Ethical considerations, permits and authorship**

*This section is applicable to all research types.*

Provide details as to who granted permissions and/or consent for the study to take place in the Methods section of your manuscript. This should include the names of all ethics boards, governmental organizations, community leaders or other bodies that provided approval for the study. If individuals provided approval refer to these people by their role or title but do not list their name(s).

Reported on page number: Page 13

If there were any deviations from the study protocol after approval was obtained please provide details of these changes in the Methods section of your manuscript.
Did this study involve local collaborators that are residents of the country where the research was conducted or members of the community studied? If you do not have any authors from said communities, please provide an explanation for this below.

Reported on page number: N/A

This study did not include co-authors who are residents of, or members of, the communities where the research was conducted. However, the research was carried out in close collaboration with local partners and community members throughout the process. Their contributions included facilitating access, supporting data collection, and providing contextual insights to ensure cultural relevance and sensitivity.

These collaborators are acknowledged in the acknowledgements section. While they are not listed as co-authors in accordance with authorship criteria, their input was essential in strengthening the validity and contextual grounding of the study.

Everyone listed as an author should meet PLOS’ criteria for authorship and all individuals who meet these criteria should be included in the author byline, rather than the acknowledgements. For further information please see the journal’s Authorship Policy.

**Human subjects research (e.g. health research, medical research, cross-cultural psychology)**

Did you obtain written informed consent from a representative of the local community or region before the research took place? How did you establish who speaks for the community? Details of written informed consent obtained from study participants should be reported separately in the Methods section of your manuscript.

Written informed consent from a single representative of the local community or region was not obtained, as no formal or universally recognized authority was identified as speaking on behalf of the entire community. Instead, access and engagement were established through collaboration with local partners and community stakeholders (e.g., local facilitators, academicians and organizations), who provided guidance on culturally appropriate procedures and supported community entry. Consent for participation was obtained individually from all study participants, as detailed in the Methods section.

How did members of the local community provide input on the aims of the research investigation, its methodology, and its anticipated outcome(s)?

Members of the local community contributed to the study through ongoing collaboration with local partners and facilitators. They were actively involved in reviewing the research protocols prior to submission to ethics committees, ensuring cultural appropriateness and feasibility. They also supported the forward and backward translation of study materials and helped refine interview and survey questions to enhance cultural relevance.

In addition, community members assisted in conducting interviews and focus groups and played important roles as translators. Their input helped ensure that the research aims, methodology, and anticipated outcomes were grounded in locally meaningful perspectives and accurately reflected community experiences.

When engaging with the local community, how did you ensure that the informed consent documents and other materials could be understood by local stakeholders?

All materials were translated into local languages using forward and backward translation procedures, with input from local collaborators to ensure accuracy and cultural appropriateness.

The wording of consent forms and study instruments was adapted to be clear, simple, and culturally relevant, avoiding technical language. Local facilitators and translators were present during the consent process to explain the study, answer questions, and confirm participants’ understanding. Where needed, information was also provided verbally to accommodate varying literacy levels. These steps helped ensure that participants could make informed and meaningful decisions about their participation.

Will the findings of the research be made available in an understandable format to stakeholders in the community where the study was conducted (e.g. via a presentation, summary report, copies of publications, etc.)? Please provide details of how this will be achieved.

Yes, the findings will be shared with stakeholders in accessible and culturally appropriate formats. A brief summary report will be prepared in relevant local languages, using clear, non-technical language. Findings will also be presented through meetings or workshops with local partners, community members, and stakeholders involved in the study. Where feasible, copies of publications will be shared with local collaborators and institutions. In addition, results will be communicated through local facilitators, who can further explain and discuss the findings within the community.

**Non-human subjects research using specimens/ animals collected as part of the study, or those housed in archival collections. Examples include archaeology, paleontology, botany and zoology.**

Did the permission you obtained from a local authority to perform the study include an agreement on access to outputs and benefit sharing? This may include procedures to enable fair distribution of the benefits and resources arising from the research performed. Please include any details of Prior Informed Consent and Benefit Sharing Agreements obtained. These may be required by field-specific regulations, for example the Convention on Biological Diversity (CBD) and the associated Nagoya Protocol.

NA

If the material used in your study was imported, please A) provide the year it was imported and B) indicate whether permits were obtained to import/export the materials used, C) provide details of any permits obtained. If this information is not available, please indicate this.

NA

If you used archival specimens, please state how the material used in your study was acquired by the institute it is held in and provide details of any permits obtained for the original excavations/ sample collection. If this information is not available, please indicate this.

NA

How was the potential cultural significance of the materials collected in your study to local communities considered in your research design? Were Indigenous peoples and/or local researchers and institutions involved with archaeological excavations / collection of specimens? If so, please provide a description of their involvement.

NA

If your manuscript includes photographs of human remains please indicate whether authors obtained permission from descendants or affiliated cultural communities to do so.

NA
