# Supplementary material for: Eastern African expressions of suffering: A new Cultural Scripts of Trauma Inventory
Source: PLoS One. 2026 Sep 25;21(9):e0358482. doi: 10.1371/journal.pone.0358482 (PMC13614634; doi:10.1371/journal.pone.0358482)
Supplement: Supplementary file 2 [file pone.0358482.s004.docx]

*S1 list of mapped cultural Scripts of Trauma Suffering: Initial pool of items*

| **No** | **Items (English)** | **0** | **1** | **2** | **3** | **4** |
| --- | --- | --- | --- | --- | --- | --- |
|  | **Cognitive and affects.** |  |  |  |  |  |
| 1 | I am a sad person |  |  |  |  |  |
| 2 | I have no more love |  |  |  |  |  |
| 3 | I am a nervous person |  |  |  |  |  |
| 4 | I no longer feel like a person like others |  |  |  |  |  |
| 5 | I feel that I am cruel |  |  |  |  |  |
| 6 | I feel that I have lost my resources for pride |  |  |  |  |  |
| 7 | I feel that I have lost my dignity |  |  |  |  |  |
| 8 | I feel I wish to revenge |  |  |  |  |  |
| 9 | I feel that I can't apologize |  |  |  |  |  |
| 10 | I feel resentment |  |  |  |  |  |
|  | **Worldview** |  |  |  |  |  |
| 11 | I feel like this world is not fair |  |  |  |  |  |
| 12 | I doubt that there is a God |  |  |  |  |  |
| 13 | I believe that people of this world are unpredictable |  |  |  |  |  |
| 14 | I feel that this world is not our home |  |  |  |  |  |
| 15 | I believe that there is no need to plan for tomorrow |  |  |  |  |  |
| 16 | I feel I must enjoy my life today unconditionally |  |  |  |  |  |
| 17 | I must be cautious about everything |  |  |  |  |  |
| 18 | I am always ready to anticipate danger |  |  |  |  |  |
| 19 | I alert the security services even for suspected incidents |  |  |  |  |  |
|  | **Interpersonal relationship** |  |  |  |  |  |
| 20 | I am glad when my enemy sees my successes |  |  |  |  |  |
| 21 | I am happy when my enemy remains under my control |  |  |  |  |  |
| 22 | I wish bad things to my enemies but I can support them |  |  |  |  |  |
| 23 | I feel that I can’t trust anyone |  |  |  |  |  |
| 24 | I feel that no one wants to support me |  |  |  |  |  |
| 25 | I feel that all people only work for their own interests |  |  |  |  |  |
| 26 | I feel people have to value who I am |  |  |  |  |  |
| 27 | I feel unhappy when no attention is given to my activities |  |  |  |  |  |
| 28 | I feel uncomfortable when people criticize what I did |  |  |  |  |  |
| 29 | I like competition with my enemies |  |  |  |  |  |
| 30 | I feel like I do not have a role model in my life |  |  |  |  |  |
| 31 | I do not feel that people are so important |  |  |  |  |  |
| 32 | I do not feel that without the support of people, I could die |  |  |  |  |  |
| 33 | I feel that I have to support people |  |  |  |  |  |
|  | **Body related** |  |  |  |  |  |
| 34 | I have sleeping problems |  |  |  |  |  |
| 35 | I have headaches |  |  |  |  |  |
| 36 | I have backaches |  |  |  |  |  |
| 37 | I have skin aches |  |  |  |  |  |
| 38 | I feel dizzy |  |  |  |  |  |
| 39 | I feel tired |  |  |  |  |  |
| 40 | I have stomach ache |  |  |  |  |  |
| 41 | I feel tingling in my skin |  |  |  |  |  |
| 42 | I feel the smell of blood |  |  |  |  |  |
| 43 | I feel smell that I cannot explain |  |  |  |  |  |
| 44 | I feel something strange in my body |  |  |  |  |  |
| 45 | I feel a temporary loss of consciousness |  |  |  |  |  |
| 46 | I feel a sensation of throbbing in the head |  |  |  |  |  |
| 47 | I feel the inability to gain weight |  |  |  |  |  |
| 48 | I have eye disease |  |  |  |  |  |
|  | **Meaning disruption** |  |  |  |  |  |
| 49 | I do not need to strive for myself |  |  |  |  |  |
| 50 | I feel like I have not succeeded in life |  |  |  |  |  |
| 51 | Since that time, I have not achieved any success. |  |  |  |  |  |
| 52 | I do not mind even if I waste time. |  |  |  |  |  |
| 53 | I do not have faith that everything is possible. |  |  |  |  |  |
| 54 | I give up easily |  |  |  |  |  |
| 55 | I do not think twice before acting |  |  |  |  |  |
| 56 | I feel like I am no longer wise |  |  |  |  |  |
| 57 | It is hard to apply what I learned from hardship. |  |  |  |  |  |
| 58 | I feel that I am no longer grateful |  |  |  |  |  |
| 59 | I no longer have my kindness |  |  |  |  |  |
| 60 | I doubt that nothing is impossible for God. |  |  |  |  |  |
| 61 | It is hard to believe in God |  |  |  |  |  |
|  | **Family Stress** |  |  |  |  |  |
| 62 | I feel that I am alone |  |  |  |  |  |
| 63 | I feel that I have an empty family |  |  |  |  |  |
| 64 | I need more family members |  |  |  |  |  |
| 65 | I feel no need to get married |  |  |  |  |  |
| 66 | I know that all people are bad |  |  |  |  |  |
| 67 | I am not able to communicate efficiently with a family member |  |  |  |  |  |
| 68 | I am aggressive towards close others |  |  |  |  |  |
| 69 | I have no positive feelings towards close others |  |  |  |  |  |
| 70 | I don't know how to manage family resources |  |  |  |  |  |
| 71 | I feel that everything is vanity |  |  |  |  |  |
