# Supplementary material for: Eastern African expressions of suffering: A new Cultural Scripts of Trauma Inventory
Source: PLoS One. 2026 Sep 25;21(9):e0358482. doi: 10.1371/journal.pone.0358482 (PMC13614634; doi:10.1371/journal.pone.0358482)
Supplement: Supplementary file 3 [file pone.0358482.s005.docx]

**Instructions**: **1. English:** The following statements describe problems that people sometimes report in response to traumatic or stressful life events. Please read each statement carefully, then circle one of the numbers to the right to indicate how much you have been bothered by that problem in the past month. Write down one event that affected you more deeply than any other, and use it as a reference point when answering the questions: e.g. genocide, childhood trauma, war, accident, imprisonment etc.

*Cultural Script of Trauma Inventory in all languages (CSTI)*

|  |  | **Score** | | | | | |
| --- | --- | --- | --- | --- | --- | --- | --- |
| **No** | **English** |  | **0** | **1** | **2** | **3** | **4** |
|  | **Cognitive** |  |  |  |  |  |  |
| **1** | I have no more love |  |  |  |  |  |  |
| **2** | I no longer feel like a person like others |  |  |  |  |  |  |
| **3** | I feel that I have lost my dignity |  |  |  |  |  |  |
|  | **Frame of mind** |  |  |  |  |  |  |
| **4** | I believe that there is no need to plan for tomorrow |  |  |  |  |  |  |
| **5** | I feel that no one wants to support me |  |  |  |  |  |  |
| **6** | I feel like I do not have a role model in my life |  |  |  |  |  |  |
|  | **Body related** |  |  |  |  |  |  |
| **7** | I have sleeping problems |  |  |  |  |  |  |
| **8** | I have headaches |  |  |  |  |  |  |
| **9** | I feel tingling in my skin |  |  |  |  |  |  |
|  | **Meaning disruption** |  |  |  |  |  |  |
| **10** | I do not need to strive for myself |  |  |  |  |  |  |
| **11** | Since that time, I have not achieved any success. |  |  |  |  |  |  |
| **12** | I do not have faith that everything is possible. |  |  |  |  |  |  |
|  | **Family stress** |  |  |  |  |  |  |
| **13** | I feel that I have an empty family |  |  |  |  |  |  |
| **14** | I am aggressive towards close others |  |  |  |  |  |  |
| **15** | I don't know how to manage family resources |  |  |  |  |  |  |

**Scoring**

Notes: *Items 2, 4, 8, 10, 14 were found to be consistent in all countries as shown by partial scalar invariance.*

**Item Scoring**: 0: Strongly Disagree; 1: Disagree; 2: Neutral; 3: Agree; 4: Strongly agree

**Total Scale Scoring**: The CSTI ranges from 0 to 60, with 0 indicating absence of CST symptoms. A score ≥2 on any subscale, especially when accompanied by clinical symptoms or functional impairment, is considered clinically significant. In clinical settings, even one CST symptom scored ≥2 may warrant clinical attention.
