# Supplementary material for: Eastern African expressions of suffering: A new Cultural Scripts of Trauma Inventory
Source: PLoS One. 2026 Sep 25;21(9):e0358482. doi: 10.1371/journal.pone.0358482 (PMC13614634; doi:10.1371/journal.pone.0358482)
Supplement: Supplementary file 4 [file pone.0358482.s006.docx]

**Complete description of qualitative dataset**

This dataset comprises qualitative data collected from trauma survivors and trauma experts through focus group discussions and individual in-depth interviews. The study explored cultural scripts of trauma in East African contexts.

Data were collected in four countries: Rwanda, Uganda, Kenya, and Tanzania. The dataset includes verbatim transcripts from focus groups and individual interviews conducted with trauma survivors and trauma-related professionals, including mental health practitioners and community-based support providers such as traditional and spiritual healers. Interviews were conducted using semi-structured guides and facilitated by trained multilingual researchers.

The dataset includes transcripts in Kinyarwanda (Rwanda), English (Uganda and Kenya), and English and Swahili (Tanzania). All audio recordings were transcribed verbatim and translated where necessary, following standardized qualitative research procedures to ensure cultural and conceptual equivalence across languages. All data were anonymized prior to analysis to protect participant confidentiality. In Rwanda participants and experts in Kenya did not allow audio recording so, the results are the written Memos.

The dataset is suitable for qualitative methodologies such as thematic analysis, framework analysis, grounded theory, and cross-cultural comparative studies.

**Metadata Fields**

- **Study design:** Qualitative multi-country study using focus group discussions and in-depth interviews
- **Study setting:** Rwanda, Uganda, Kenya, Tanzania
- **Study population:** Trauma survivors and trauma experts (including mental health professionals, spiritual and traditional healers)
- **Data collection methods:** Semi-structured focus group discussions and individual interviews
- **Data type:** Qualitative textual transcripts
- **Languages:** Kinyarwanda, English, Swahili
- **Number of countries:** 4
- **Data format:** Text transcripts .pdf
- **Unit of analysis:** Individual interview transcripts and focus group discussion transcripts
- **Time period of data collection:** March 2024-September 2025.
- **Sampling strategy:** Purposive and snowball sampling
- **Ethical approval:** Approved by This research was approved by Ethics Committee of the University of Rwanda, College of Medicine and Health Sciences, approval nr. 396/CMHSIRB/2023, Ethics Committee of the Daystar University Kenya, approval nr. DU-ISERC/09/05/2023/00086, ETH Zurich ethics committee Nr. EK 2023-N-308, University of Dar es Salaam ethical Committee Ref. No: AB3/12(B) and Makerere University School of Health Sciences ethical committee MAKSHSREC-2025-1903.
- **Consent:** Written informed consent obtained from all participants
- **Anonymization:** All transcripts de-identified prior to analysis but the dataset has sensitive information of the participants.
- **Funding source:** No funding source
- **Data access conditions:** The data are available to the first author on the following email: [celestin.mutuyimana@psychologie.uzh.ch](mailto:celestin.mutuyimana@psychologie.uzh.ch) or [clestino06@gmail.com](mailto:clestino06@gmail.com) , and to the ethical committee contact person in Tanzania Dr. Nkuba George Mabula [mabula.nkuba@duce.ac.tz](mailto:mabula.nkuba@duce.ac.tz) and to the contact person in Kenya Prof. Stephen Asatsa, [steveasatsa@gmail.com](mailto:steveasatsa@gmail.com) and representative of College of Medicine and Health Science fsunday@khi.ac.rw
- **Software used for analysis:** MAXQDA. Transcripts in other languages were analyzed in that language only codes were translated
- **File organization:** All transcripts are in the same folder
