# Supplementary material for: Eastern African expressions of suffering: A new Cultural Scripts of Trauma Inventory
Source: PLoS One. 2026 Sep 25;21(9):e0358482. doi: 10.1371/journal.pone.0358482 (PMC13614634; doi:10.1371/journal.pone.0358482)
Supplement: Supplementary file 5 [file pone.0358482.s007.docx]

**Review of *Eastern African Expressions of Suffering: a new Cultural Scripts of Trauma Inventory***

**Review summary:** This manuscript aimed to validate a measure that captures cultural scripts of trauma in four Eastern African countries. The authors did a great job executing a qualitative and quantitative approach to building and validating the measure across both individuals who have experienced trauma and local trauma experts. However, some nuance throughout the paper is warranted to gain a better sense of cultural context, execution of the study, and interpretation of results. Some English grammar and edits are needed as well.

**Introduction:**

Pg 3, lines 4-6: Please provide examples of the most prevalent types of trauma to help provide contextual information about these traumatic experiences.

Pg 3, lines 7-12: It would be helpful to add in more information and context about the culture around seeking mental health support for trauma, perhaps highlighting the gap that needs to be filled with having a tool to increase the recognition of PTSD symptoms.

Pg 3, lines 16-17: Is there any literature to suggest differences in trauma or response to trauma across East African countries? If so, I think this is important to highlight and can be tied into the discussion section.

**Methods:**

Pg 5, lines 46-48: How were the inclusion criteria screened for to determine eligibility (i.e., experience of trauma-related symptoms, perception of cultural values, norms, beliefs)?

Pg 5, line 55: Where were the in-person focus groups held? And how many participants in each group?

Pg 5, line 55: Please describe informed consent process.

Pg 6, line 70: It would be helpful to clarify which measures were used in which part of the study (i.e., interview guide for qualitative component).

Pg 6, line 72: Please expand on what central symptoms refer to.

Pg 6, line 74: It’s a bit confusing to have the CSTI listed here. I would either be super clear that it was being assessed in this paper or remove it from this section. This should also follow the same format as the other measures, so it should just be the construct in the header.

**Results:**

Pg 9: The N should have an equal sign not a dash in tables 1 and 2.

Pg 10: Table 2 should say participant characteristics and not trauma expert characteristics.

Pg 11: More information on how interview data was converted into these 71 items. How was this organized?

Pg 14: It may make sense to have demographic characteristics displayed in the same way for the qualitative and quantitative studies to improve clarity and consistency (i.e., table, written out, or both).

Pg 14: The translation and item content validity assessment section is a bit confusing because it describes what’s in Table 3, which comes earlier in the manuscript. Consider reorganizing.

Pg 15-16: In Table 4, items 2 and 13 are hard to understand in English. Due to the likely cultural relevance of them, I would just highlight what they aim to capture to provide clarity for readers who are unfamiliar with East African culture.

Pg 17: Please clarify the justification as to why the questionnaire is reliable and valid in Kenya despite the results.

**Discussion:**

Pg 21: The authors highlight the need for non-stigmatizing spaces. It would be helpful to see an integration of past studies that have developed interventions to address trauma (or other mental health symptoms) in a similar manner and context.

Pg 21: It would be helpful to situate these findings in the context of the current literature to emphasize the need for a measure like this, as well as the implications for it.

Pg 21: Why do the authors think the CFA fit for Kenya was less satisfactory? Please include.
