# Supplementary material for: Eastern African expressions of suffering: A new Cultural Scripts of Trauma Inventory
Source: PLoS One. 2026 Sep 25;21(9):e0358482. doi: 10.1371/journal.pone.0358482 (PMC13614634; doi:10.1371/journal.pone.0358482)
Supplement: Supplementary file 6 [file pone.0358482.s008.docx]

**Dear reviewers and editor,**

We are sincerely grateful for the comments you provided, which helped to considerably improve the manuscript. We have responded to all the questions raised and addressed them in the revised manuscript. Below is the table with details.

| Questions | Answers | Page |
| --- | --- | --- |
| **Reviewer 1** | | |
| 1.I am not a statistics expert, so I can't confirm the accuracy of the statistics performed in this manuscript. I believe the authors are not allowed to share the data from this study, but it would be helpful to have more qualitative data incorporated into the manuscript if possible. | **Thank you for your comment. All data necessary for the development of this inventory is included in the manuscript, with additional data provided in the supplementary materials.** | S1 |
| The English in the manuscript is acceptable, but some editing is required. | **Thank you for your comment. We have revised the manuscript accordingly.** | All |
| **Reviewer 2** | | |
| This manuscript presents the development and validation of the Cultural Scripts of Trauma Inventory (CSTI), a new measure intended to capture culturally specific trauma-related expressions in East African populations (Rwanda, Uganda, Tanzania, Kenya). The study uses a mixed-methods design, beginning with qualitative focus groups to generate culturally grounded items, followed by a large quantitative validation study (N = 1,208). The research addresses an important gap in trauma research by attempting to move beyond Western-centric PTSD frameworks and incorporating culturally contextualized symptoms.  Overall, the manuscript tackles an important and timely topic, and the large multi-country sample and mixed-method approach are clear strengths. Additionally, incorporating the voices of individuals with lived experiences, as well as traditional and spiritual healers, is another strength. However, several methodological, conceptual, and reporting issues limit the clarity and robustness of the conclusions. In its current form, the manuscript would benefit from revision. | **Thank you for your positive evaluation and constructive feedback. We have substantially improved the manuscript based on your comments.** | All |
| I see several strengths of this manuscript:  The study addresses a significant limitation in global trauma research: the dominance of Western PTSD frameworks and the underrepresentation of culturally specific expressions of distress. Developing a culturally sensitive measure is highly relevant for global mental health. Therefore, this manuscript may provide an important conceptual contribution to the research field.  The qualitative → quantitative sequential design is appropriate for scale development. Using focus groups to generate culturally grounded items strengthens ecological validity.  The quantitative validation sample is relatively large (N = 1,208 across four countries), which increases statistical power and supports factor analytic procedures. Including multiple East African countries improves generalizability compared to single-country scale development and supports a cross—cultural approach. The authors attempt to apply standard psychometric procedures, including exploratory factor analysis, confirmatory factor analysis, measurement invariance, reliability analyses, convergent/divergent validity. These are appropriate methods for instrument validation. | **Thank you for your positive and much appreciated feedback. We agree that the manuscript is important for global mental health and have used your comments to further strengthen it.** | NA |
| Conceptual clarity of “Cultural Scripts of Trauma”:  The concept of Cultural Scripts of Trauma (CST) is central to the paper but remains insufficiently defined and theoretically grounded. While the concept is briefly introduced, it is not clearly differentiated from related constructs such as idioms of distress or cultural syndromes, which leaves the concept somewhat vague. The introduction would benefit from clarifying how CST differs from idioms of distress and how it relates to the DSM-5 framework of cultural concepts of distress. The introduction should include some basic literature on cultural idioms of distress, the cultural adaptations in DSM-5 and ICD-11, transcultural psychiatry and global mental health.  The manuscript could also benefit from engaging more explicitly with the cultural considerations reflected in the ICD-11 framework for PTSD and Complex PTSD. Evidence supporting ICD-11 highlights the cross-cultural validity of CPTSD in both Western and non-Western contexts, identifies low self-compassion as a consistent marker across cultures, and acknowledges that while PTSD is globally recognized, the expression of symptoms may vary across cultural settings. In this context, CST—and particularly the CSTI—could be positioned as a useful tool for capturing such culturally shaped differences in symptom expression. | **We appreciate the comment. The current manuscript has incorporated the text that makes distinction between CST and idioms of distress and the necessity of CST.** | Page 4-6, Line 24-69 |
| Sampling bias in quantitative study:  Participants were recruited via social media platforms (Facebook, WhatsApp, Instagram, X). This creates some bias: high education level (≈60% bachelor’s degree), urban skew (≈73% urban) and likely younger, digitally connected populations. This contradicts the claim that the measure reflects broader East African cultural expressions. The authors should acknowledge the selection bias and the limited representation of rural or less educated populations in the limitation section. This is particularly important because trauma experiences may be often more prevalent in non-urban populations. | **Thank you for your comments. We have addressed this issue in the limitations section. However, recruitment was not conducted exclusively online across all countries; some sites used a mixed approach combining online and in-person recruitment. In addition, while the quantitative sample is relatively young, the qualitative sample is more age diverse. More broadly, East African populations are predominantly young, with approximately 85–91% of individuals under the age of 45 across the included countries.**  **Furthermore, although some participants are students and primarily based in urban areas, this reflects the structure of higher education systems, where most universities are in cities. Nevertheless, many participants maintain strong ties to rural areas or have lived experience of rural contexts, ensuring that their perspectives are not limited to urban settings**. | Page 25, line 478-488 |
| Item development process unclear:  The development of the 15-item CSTI is not completely clear to me. How were the initial 98 items generated? Were items derived directly from qualitative data? Were coders independent? The authors could provide a clearer development workflow, e.g. qualitative coding, item generation, expert review, pilot testing and item reduction criteria. The decision rules in reduction from 98 to 71 to 15 items could also be better described. Reducing 71 culturally rich items to only 15 items may also remove meaningful cultural nuance and may even oversimplify the construct. Moreover, only three items per factor are few and might be statistically fragile. The authors could justify these issues. | **We thank the reviewer for this important request for clarification regarding the development of the CST Inventory (CSTI). The description of item generation is now included in this manuscript**  **We acknowledge that reducing 71 culturally rich items to a shorter scale may involve loss of nuance. However, the CSTI is intended as a brief, cross-culturally applicable screening instrument, while the full qualitative dataset and extended item pool remain available for preserving cultural richness and supporting further qualitative and mixed-methods analyses. The aim was therefore to balance cultural depth with psychometric robustness and usability across contexts** | Page 8, line 121-142 |
| *Factor analytic procedure is not fully appropriate:*  The manuscript uses EFA and CFA on the same dataset. Although the authors mention two subsamples, it is unclear how the split was conducted and whether the EFA and CFA samples were independent. This could introduce overfitting. The authors should clarify the exact subsample sizes and whether EFA and CFA were conducted on separate datasets. Moreover, the CFA results show poor model fit for Kenya. However, the manuscript dismisses this issue too quickly and still recommends the scale for Kenya. The authors should investigate why Kenya differs, test measurement invariance more thoroughly and consider cultural heterogeneity | **We thank the reviewer for this important comment. Now clear details are mentioned in the manuscript. The total sample was randomly divided into two equal and independent subsamples, with one used for Exploratory Factor Analysis (EFA) and the other for Confirmatory Factor Analysis (CFA). This split-sample approach is a commonly recommended strategy in scale development when the overall sample size is sufficiently large, as it allows for cross-validation of the factor structure while minimizing the risk of overfitting (e.g., Worthington & Whittaker, 2006).**  **Regarding the Kenyan sample, we acknowledge that the entire model fit did not reach conventional thresholds. However, Comparative Fit Index CFI is relatively good and psychometric properties, including factor loadings, internal consistency, and overall factor structure, were satisfactory. This suggests that the scale retains acceptable structural validity in this context. At the same time, we agree that this discrepancy may reflect cultural heterogeneity or contextual differences in symptom expression. This was mentioned in discussion section.** | Page 12, line 217-222, Page 23, line 443-447 |
| Interpretation sometimes exceeds the evidence: Several claims in the discussion are stronger than the results support. For example,  the paper claims the CSTI enables “culturally informed diagnosis and treatment.” However, the study did not test clinical outcomes, and the sample was not clinical. The authors might tone down these claims. | **We thank the reviewer. Although we did not find in the manuscript where we said that CSTI enables culturally informed diagnosis and treatment; we revised the manuscript to tone down these claims and clarify that the CSTI is intended to inform culturally sensitive assessment, with implications for diagnosis and treatment to be tested in future clinical research.** | all |
| The manuscript suggests that one item score ≥2 may be clinically significant. This is not empirically justified. Diagnostic thresholds might need clinical validation. | **We thank the reviewer for this important point. We agree that the threshold of ≥2 should not be interpreted as a diagnostic cutoff. In the present study, this value was used as a *pragmatic indicator of symptom presence and salience*, consistent with common practice in symptom inventories, where scores above minimal levels are often considered indicative of meaningful experience rather than absence of symptoms (e.g., Streiner & Norman, 2008). We fully agree that clinically meaningful cutoffs require validation in clinical samples, including sensitivity and specificity analyses, which remain an important direction for future research. This was mentioned in limitation section.** | Page 25, 485-488 |
| The manuscript contains many grammatical errors. These appear throughout the manuscript. The paper requires professional language editing.  • “culturally scripts of trauma sufferings”  • “the confirmatory factor analysis confirm”  • “sequalae”  • „Survivors in Namibia was perceiving their sufferance as something standing inside“-> e.g.: „Survivors in Namibia perceived their suffering as something that existed within them.“  • „exeperts“  • e.g. lines 5, 6, 23: Spaces are missing or duplicated.  Some sentences sound awkward in academic English:  • Line 11: „Those sufferings are in a new theory investigated as Cultural 12 Scripts of Trauma (CST)“ -> e.g.: „These forms of suffering are conceptualized in a new theoretical framework as Cultural Scripts of Trauma (CST).“  • Line 91: „The 20-item of Clinical Aspects of Historical Trauma Questionnaire concerns cultural patterns of men-made collective trauma, prevalent in Eastern Africa.“ -> e.g. „The 20-item Clinical Aspects of Historical Trauma Questionnaire assesses cultural patterns of man-made collective trauma prevalent in Eastern Africa. | **We appreciate the reviewer’s observation. We have corrected the typographical errors, and the manuscript has been proofread by a native English speaker.** | All |
| Please clarify why Rwanda was not included in the quantitative part of the study. | **Thank you for the question. However, Rwanda was included in all quantitative analysis.** |  |
| Please indicate whether the questionnaires used in this study (other than the CSTI, which was developed here) have been validated for the respective cultural and linguistic contexts in which they were applied (Uganda, Tanzania, Rwanda, and Kenya). | **Thanks for the question: Only Clinical Aspects of Historical Trauma and International Trauma Questionnaire and ITQ were used before in these samples, but others were translated and culturally adapted before use and have shown good reliability.** | Page 9, line 146-151 |
| Please list the subscales of the Clinical Aspects of Historical Trauma Questionnaire in the methodology section. In addition, please clarify what is meant by the term “clinical aspects of historical trauma.” | **Thank you for your comment. The subscales have been added to the manuscript. The Clinical Aspects of Historical Trauma is the name of questionnaire developed to assess the clinical manifestations of Historical Trauma.** | Page 10, line 181-183 |
| Under which ethical approvals was data collection in Tanzania and Uganda conducted? Please clarify why local ethical approval appears to have been obtained only for Kenya and Rwanda. | **Thank you for your question. The ethics committee in Uganda previously reviewed the study protocol along with ethics approvals from ETH Zurich, Kenya, and Rwanda, and approved the study in collaboration with local partners. Following your comment, we additionally obtained and included the formal ethics approvals from Tanzania and Uganda.** | Page 13, Line 234-235 |
| Participants were included if they had “past traumatic experiences.” However, the type and severity of trauma are not clearly operationalized. The authors should clarify how trauma exposure was defined and assessed. | **Thank you for the comment. Traumatic experiences were defined as exposure to man-made traumatic events, childhood trauma, or accidents. These categories were listed in advance to ensure participants clearly understood the types of events being referred to when responding.** | S1 (Interview guide) |
| Several items in the scale appear to be unrelated to trauma. For example, items such as “I am proud of my achievements” or “I believe everything is possible” might resemble general well-being or personal growth items rather than trauma-related symptoms. The conceptual rationale for including such items should be clarified | **Thank you very much for this helpful observation. This was a wording issue in the manuscript; however, the questions were originally phrased in the negative form, and we have now corrected this in the revised manuscript.** | S3 |
| East Africa is culturally diverse. Combining four countries may obscure important differences in religion, conflict history, and trauma exposure. The manuscript should discuss cross-cultural variability more critically. | **Thank you for the comment. We agree with this point and have addressed it in the limitations section.**  **However, participants came from diverse backgrounds and subcultures. Nevertheless, consistent with Anthony J. Marsella’s (1988) definition of culture as shared values, meanings, and ways of living, East African communities broadly share relational cultural values such as Ubuntu, collectivism, and humanism.** | Page 25, Line 477-479 |
| Some tables are difficult to interpret, for example Table 7 (HTMT values) and Table 6 (item analysis). Clearer formatting and labeling would improve readability. | **Thank you for this observation. The tables have now been reformatted to improve clarity** | See all Tables |
| I do not understand the age categories used in the manuscript. For example, the group’s 29–44 and 41–55 appear to overlap. Please clarify the rationale for dividing the sample into these categories. I also do not understand the division into three age groups presented in Table 2. Please explain why these specific age groupings were chosen instead of reporting mean age and standard deviation. Relatedly, the manuscript does not report standard deviations (SDs) for several variables. Please clarify why these are not included, as SDs would help readers better understand the distribution of the data. | **Thank you for this comment. We have now reported age as a continuous variable, including the mean and standard deviation.** | Page 13-14 Line 242-257 |
| Discussion:  • The manuscript states: “Participants described how traumatic experiences extend beyond the individual level to affect the entire family.” This statement seems to imply that such family-level effects are specific to people in Sub-Saharan Africa. However, although these dynamics may not be explicitly reflected in diagnostic frameworks such as ICD-10 or ICD-11, this does not mean that patients in Europe do not experience similar family-level consequences of trauma. A more balanced discussion would be helpful. Are there studies from European contexts examining how PTSD-related suffering extends beyond the individual to affect the family system? From my clinical experience, such family dynamics are also highly relevant in European settings. | **Thank you for this appreciated comment. We agree that the effects of trauma extend beyond the family level across many contexts. In the revised manuscript, we have included a more balanced discussion of these broader family impacts.** | Page 22, line 421-428 |
| The authors refer to PTSD and cPTSD in the introduction, yet this distinction is not clearly reflected in the discussion of cultural scripts. It would be helpful to clarify whether the distinction between simple and complex post-traumatic experiences plays a role within the concept of cultural scripts. This may be particularly relevant for aspects such as interpersonal relationship difficulties, which are central features of complex trauma presentations. | **Thank you for this comment. In the discussion we have included the section showcasing the differences and some similarities between PTSD, CPTSD and CST** | Page 24, Line 459-472 |
| **Reviewer 3** | | |
| I congratulate the authors to their manuscript. It advances the discussion of the PTSD concept considerably. The challenge of this manuscript is that is very complex and word limit needs to be balanced with reporting of essential information. I tried to provide some comments on how to further develop this manuscript. | **Thank you very much for your positive feedback. We greatly appreciate your comments, which helped us substantially improve the manuscript.** |  |
| Abstract:  - It is confusing for a reader the fact that the pre-CSTI had 71 items is first presented in Methods and then, again presented as if a new information in Results. It might be sufficient to present the number of items in Results. | **Thank you for this comment. Now we have deleted the number of items in the method section.** | Abstract |
| Introduction:  - Line 33 ff: Please state here the aims and if relevant hypotheses of the qualitative and quantitative studies | **Thanks for your comments, the aim of the study is now presented. The hypotheses are not relevant for this study.** | Page 6, Line 82-84 |
| Methods:  - Line 43 ff: Some aspects of the qualitative study remain vague, e.g. who conducted the interviews and the analysis, the languages and settings that were involved etc. How was your approach in the analysis re the different languages, e.g. did you translate all scripts to and do the qualitative work in one of the languages? | **We appreciate that comment. The interviews were conducted by the first author together with a local psychologist in each country. They were carried out in the participants’ preferred languages: Kinyarwanda in Rwanda; English in Uganda; and both English and Swahili in Kenya and Tanzania. All interviews were audio-recorded and transcribed verbatim.**  **For data analysis, interviews conducted in Kinyarwanda and Swahili were coded independently by two local coders fluent in the respective languages. Coding was performed in the original language to preserve cultural and linguistic nuances, and the resulting codes were subsequently translated into English. Interviews conducted in English were also coded independently by two researchers and additionally reviewed by a local coder to ensure contextual accuracy. Following the initial coding process, the first author conducted a comprehensive review of all codes in collaboration with a local psychologist. Any discrepancies were systematically discussed until consensus was reached, resulting in a finalized and culturally validated coding framework. This section was included in the current manuscript** | Page 8, Line 118-134 |
| - Line 62 – 65: Please state clearly for what reason you used social media. | **Thank you for your comment. We have clarified this rationale in the manuscript. As this was an online study, social media provided an efficient way to recruit diverse participants across settings.** | Page 9, Line 148-151 |
| - Line 62 ff: It remains vague how the quantitative data assessment took place, e.g. how participants filled out the questionnaires, languages used etc. | **Thank you for the comment. The process is detailed. The participants filled out online or in person survey and in local language or English.** | Page 9, Line 152-156 |
| - Line 69 ff: It would be helpful to reorder the description of your measures and to assign them to the qualitative and quantitative study. | **Thank you for the comment. We have reordered the description to improve clarity and readability.** | See tools and Interview guide |
| - Line 79 ff: The description of your interview protocol guide is very brief. Please refer to the theoretical approach you followed, the way your interview guidelines were developed etc. The qualitative work needs to be better described, i.e. what were the guiding questions for the coders. The systematic how items were developed based on qualitative data remains unclear, please elaborate on that. | **Thanks for this important comment. Now we have extended the interview protocol guide section, and we have included further details mentioned. The methods section is elaborated sufficiently.** | Page 6-7, Line 94-99 |
| - Line 74 ff: It is confusing that you present the result of the study, the final CSTI, in the methods section as if it was employed like the other questionnaires. I recommend that you describe the final CSTI at the end of the results section. | **Thank you for your comment. In this manuscript is mentioned in parathesis that it is subject to this study.** | Page 10, line 162 |
| - Line 82 ff: Please provide some information on whether the employed quantitative instruments can be considered culturally appropriate and on how you made sure that validated and comparable language versions were available. | **Thanks for your comment. It helped in adding the section of translation and cultural adaptation of instruments** | Page 9, line 146-151 |
| - Please find a way to clearly name the two meanings of discriminant validity in your study. | **In this study, construct validity was addressed in terms of (1) the structural representation of the construct and (2) the differentiation between constructs.** | NA |
| Results:  Table 1 and Table 2: The reporting of the mean of the variable age is not adequate in this table format, especially as the coefficient is in the column “Frequency”; furthermore, the SD needs to be reported, too. I recommend to restructure the table or report it in the text. | **Thank you for the comments. Have reported in wording this tables and SD are mentioned.** | Page 13-14, line 243-257 |
| - After Line 153 the line count stopped. | **Sorry for this error.** | NA |
| Table 3: Very important information; but it might be moved to an electronic annex. | **Thanks for the comments. Now the Table is in Supporting materials** | S3 |
| - Interpretation of the CSTs in the different categories: E.g. you write re the third category “These accounts illustrate how, in contrast to findings from Western contexts, trauma in East African settings can deeply disrupt family functioning and contribute to a loss of hope within the family unit.” These statements seem to better fit into the discussion section. E.g. concerning the third category, it is a matter of discussion whether the disruption of families and the loss of hope are specific or universal cultural phenomena. | **Thank you for mentioning this. We have included a short paragraph in the discussion section mentioning the difference and universality of trauma symptoms at family level** | Page 22, line 421-428 |
| - EFA factor extraction: Usually, you first perform the tests that guide you on factor number and then you perform an EFA with this number of factors. Please justify your approach. | **The method is clearly explained in the manuscript. We followed established scientific procedures for factor retention in EFA by combining multiple complementary criteria, including eigenvalues, parallel analysis, scree plot inspection, variance explained, item loading patterns, and conceptual interpretability. Although parallel analysis suggested up to eight factors, the higher-order solutions showed minimal additional variance, unstable cross-loadings, and limited conceptual distinctiveness. Therefore, consistent with recommended psychometric practice, we retained the more parsimonious and interpretable five-factor solution.** | Page 16-17, line 319-333 |
| - Sample used for EFA and CFA: Did you use the same sample for both methods? Usually separate samples are recommended, e.g. to split the original sample (2/3 vs 1/3). Please justify your approach. | **Thank you for this comment. We appreciated it and we have revised the text in methods section showing how the CFA was done.** **The total sample was randomly divided into two independent subsamples. One subsample was used for Exploratory Factor Analysis (EFA), and the second subsample was used for Confirmatory Factor Analysis (CFA). This split-sample (50%/50%) approach is commonly recommended in scale development when the sample size is sufficiently large, as it allows cross-validation of the factor structure while reducing the risk of overfitting.** | Page 12, line 216-212 |
| - Table 6: The table and its information is difficult to understand, please clarify. Especially the meaning of the coefficients should be explained (e.g. in the methods section), e.g. “Item alpha value” vs “alpha” and why in “Dimension” TS and SS are mentioned; what means “general reliability”? | **We appreciate the comments. Based on it we have removed the repetition and used only the indices for CFA sample.** | See Table |
| - Table 7: please provide the meaning of the lines. | **Thanks for the comments. We have made the table clearer** | See Table |
| Discussion:  - The discussion would profit from the point of view that the categories of CSTs detected in the qualitative study also relate to the concepts behind the DSM symptom categories. The universal vs. specific cultural nature of symptoms needs to be commented on. | **We appreciate this comment. The discussion section is included** | Page 24, line 459-472 |
| Typos and grammar  - Line 12 “was”  - Line 44 verb missing  - Line 63 verb missing  - Line 150: Please write “Qualitative results” | **Thanks very much for these observations. We have corrected all of these typos.** | All |

Thanks again for the comments
