# Supplementary material for: Eastern African expressions of suffering: A new Cultural Scripts of Trauma Inventory
Source: PLoS One. 2026 Sep 25;21(9):e0358482. doi: 10.1371/journal.pone.0358482 (PMC13614634; doi:10.1371/journal.pone.0358482)
Supplement: Peer review history

# Peer review: Decision Letter 0

**DOI:** 10.1371/journal.pone.0358482.r001

**Authors:** Inga Schalinski

24 Mar 2026

Dear Dr. Mutuyimana,

Thank you for submitting your manuscript to PLOS ONE. After careful consideration, we feel that it has merit but does not fully meet PLOS ONE’s publication criteria as it currently stands. Therefore, we invite you to submit a revised version of the manuscript that addresses the points raised during the review process.

Please submit your revised manuscript by May 08 2026 11:59PM. If you will need more time than this to complete your revisions, please reply to this message or contact the journal office at plosone@plos.org. When you're ready to submit your revision, log on to https://www.editorialmanager.com/pone/ and select the 'Submissions Needing Revision' folder to locate your manuscript file.

- A letter that responds to each point raised by the academic editor and reviewer(s). You should upload this letter as a separate file labeled 'Response to Reviewers'.
- A marked-up copy of your manuscript that highlights changes made to the original version. You should upload this as a separate file labeled 'Revised Manuscript with Track Changes'.
- An unmarked version of your revised paper without tracked changes. You should upload this as a separate file labeled 'Manuscript'.

If you would like to make changes to your financial disclosure, please include your updated statement in your cover letter. Guidelines for resubmitting your figure files are available below the reviewer comments at the end of this letter.

If applicable, we recommend that you deposit your laboratory protocols in protocols.io to enhance the reproducibility of your results. Protocols.io assigns your protocol its own identifier (DOI) so that it can be cited independently in the future. For instructions see: https://journals.plos.org/plosone/s/submission-guidelines#loc-laboratory-protocols. Additionally, PLOS ONE offers an option for publishing peer-reviewed Lab Protocol articles, which describe protocols hosted on protocols.io. Read more information on sharing protocols at https://plos.org/protocols?utm_medium=editorial-email&utm_source=authorletters&utm_campaign=protocols.

As the corresponding author, your ORCID iD is verified in the submission system and will appear in the published article. PLOS supports the use of ORCID, and we encourage all coauthors to register for an ORCID iD and use it as well. Please encourage your coauthors to verify their ORCID iD within the submission system before final acceptance, as unverified ORCID iDs will not appear in the published article. *Only*  the individual author can complete the verification step; PLOS staff *cannot*  verify ORCID iDs on behalf of authors.

We look forward to receiving your revised manuscript.

Kind regards,

Inga Schalinski

Academic Editor

PLOS One

Journal Requirements:

When submitting your revision, we need you to address these additional requirements.

1. Please ensure that your manuscript meets PLOS ONE's style requirements, including those for file naming. The PLOS ONE style templates can be found at

https://journals.plos.org/plosone/s/file?id=wjVg/PLOSOne_formatting_sample_main_body.pdf and

https://journals.plos.org/plosone/s/file?id=ba62/PLOSOne_formatting_sample_title_authors_affiliations.pdf

2. Please include a complete copy of PLOS’ questionnaire on inclusivity in global research in your revised manuscript. Our policy for research in this area aims to improve transparency in the reporting of research performed outside of researchers’ own country or community. The policy applies to researchers who have travelled to a different country to conduct research, research with Indigenous populations or their lands, and research on cultural artefacts. The questionnaire can also be requested at the journal’s discretion for any other submissions, even if these conditions are not met.  Please find more information on the policy and a link to download a blank copy of the questionnaire here: https://journals.plos.org/plosone/s/best-practices-in-research-reporting. Please upload a completed version of your questionnaire as Supporting Information when you resubmit your manuscript.

3. We note that the grant information you provided in the ‘Funding Information’ and ‘Financial Disclosure’ sections do not match.

When you resubmit, please ensure that you provide the correct grant numbers for the awards you received for your study in the ‘Funding Information’ section.

4. We note that you have indicated that there are restrictions to data sharing for this study. For studies involving human research participant data or other sensitive data, we encourage authors to share de-identified or anonymized data. However, when data cannot be publicly shared for ethical reasons, we allow authors to make their data sets available upon request. For information on unacceptable data access restrictions, please see http://journals.plos.org/plosone/s/data-availability#loc-unacceptable-data-access-restrictions.

Before we proceed with your manuscript, please address the following prompts:

a) If there are ethical or legal restrictions on sharing a de-identified data set, please explain them in detail (e.g., data contain potentially identifying or sensitive patient information, data are owned by a third-party organization, etc.) and who has imposed them (e.g., a Research Ethics Committee or Institutional Review Board, etc.). Please also provide contact information for a data access committee, ethics committee, or other institutional body to which data requests may be sent.

b) If there are no restrictions, please upload the minimal anonymized data set necessary to replicate your study findings to a stable, public repository and provide us with the relevant URLs, DOIs, or accession numbers. Please see http://www.bmj.com/content/340/bmj.c181.long for guidelines on how to de-identify and prepare clinical data for publication. For a list of recommended repositories, please see https://journals.plos.org/plosone/s/recommended-repositories. You also have the option of uploading the data as Supporting Information files, but we would recommend depositing data directly to a data repository if possible.

Please update your Data Availability statement in the submission form accordingly.

5. If the reviewer comments include a recommendation to cite specific previously published works, please review and evaluate these publications to determine whether they are relevant and should be cited. There is no requirement to cite these works unless the editor has indicated otherwise.

[Note: HTML markup is below. Please do not edit.]

Reviewers' comments:

Reviewer's Responses to Questions

**Comments to the Author**

1. Is the manuscript technically sound, and do the data support the conclusions?

Reviewer #1: Yes

Reviewer #2: Yes

Reviewer #3: Yes

**********

2. Has the statistical analysis been performed appropriately and rigorously?

Reviewer #1: I Don't Know

Reviewer #2: Yes

Reviewer #3: Yes

**********

3. Have the authors made all data underlying the findings in their manuscript fully available?

The PLOS Data policy

Reviewer #1: Yes

Reviewer #2: Yes

Reviewer #3: Yes

**********

4. Is the manuscript presented in an intelligible fashion and written in standard English?

Reviewer #1: Yes

Reviewer #2: No

Reviewer #3: Yes

**********

Reviewer #1: I am not a statistics expert, so I can't confirm the accuracy of the statistics performed in this manuscript. I believe the authors are not allowed to share the data from this study, but it would be helpful to have more qualitative data incorporated into the manuscript if possible.

The English in the manuscript is acceptable, but some editing is required.

Reviewer #2: Peer Review Report

Manuscript PONE-D-25-53823 - Eastern African Expressions of Suffering: a new Cultural Scripts of Trauma Inventory

Overall Evaluation

This manuscript presents the development and validation of the Cultural Scripts of Trauma Inventory (CSTI), a new measure intended to capture culturally specific trauma-related expressions in East African populations (Rwanda, Uganda, Tanzania, Kenya). The study uses a mixed-methods design, beginning with qualitative focus groups to generate culturally grounded items, followed by a large quantitative validation study (N = 1,208). The research addresses an important gap in trauma research by attempting to move beyond Western-centric PTSD frameworks and incorporating culturally contextualized symptoms.

Overall, the manuscript tackles an important and timely topic, and the large multi-country sample and mixed-method approach are clear strengths. Additionally, incorporating the voices of individuals with lived experiences, as well as traditional and spiritual healers, is another strength. However, several methodological, conceptual, and reporting issues limit the clarity and robustness of the conclusions. In its current form, the manuscript would benefit from revision.

I see several strengths of this manuscript:

The study addresses a significant limitation in global trauma research: the dominance of Western PTSD frameworks and the underrepresentation of culturally specific expressions of distress. Developing a culturally sensitive measure is highly relevant for global mental health. Therefore, this manuscript may provide an important conceptual contribution to the research field.

The qualitative → quantitative sequential design is appropriate for a scale development. Using focus groups to generate culturally grounded items strengthens ecological validity.

The quantitative validation sample is relatively large (N = 1,208 across four countries), which increases statistical power and supports factor analytic procedures.

Including multiple East African countries improves generalizability compared to single-country scale development and supports a cross—cultural approach.

The authors attempt to apply standard psychometric procedures, including exploratory factor analysis, confirmatory factor analysis, measurement invariance, reliability analyses, convergent/divergent validity. These are appropriate methods for instrument validation.

However, several major concerns remain, including the following:

Conceptual clarity of “Cultural Scripts of Trauma”:

The concept of Cultural Scripts of Trauma (CST) is central to the paper but remains insufficiently defined and theoretically grounded. While the concept is briefly introduced, it is not clearly differentiated from related constructs such as idioms of distress or cultural syndromes, which leaves the concept somewhat vague. The introduction would benefit from clarifying how CST differs from idioms of distress and how it relates to the DSM-5 framework of cultural concepts of distress. The introduction should include some basic literature on cultural idioms of distress, the cultural adaptations in DSM-5 and ICD-11, transcultural psychiatry and global mental health.

The manuscript could also benefit from engaging more explicitly with the cultural considerations reflected in the ICD-11 framework for PTSD and Complex PTSD. Evidence supporting ICD-11 highlights the cross-cultural validity of CPTSD in both Western and non-Western contexts, identifies low self-compassion as a consistent marker across cultures, and acknowledges that while PTSD is globally recognized, the expression of symptoms may vary across cultural settings. In this context, CST—and particularly the CSTI—could be positioned as a useful tool for capturing such culturally shaped differences in symptom expression.

Sampling bias in the quantitative study:

Participants were recruited via social media platforms (Facebook, WhatsApp, Instagram, X). This creates some bias: high education level (≈60% bachelor’s degree), urban skew (≈73% urban) and likely younger, digitally connected populations. This contradicts the claim that the measure reflects broader East African cultural expressions. The authors should acknowledge the selection bias and the limited representation of rural or less educated populations in the limitation section. This is particularly important because trauma experiences may be often more prevalent in non-urban populations.

Item development process unclear:

The development of the 15-item CSTI is not completely clear to me. How were the initial 98 items generated? Were items derived directly from qualitative data? Were coders independent? The authors could provide a clearer development workflow, e.g. qualitative coding, item generation, expert review, pilot testing and item reduction criteria. The decision rules in reduction from 98 to 71 to 15 items could also be better described. Reducing 71 culturally rich items to only 15 items may also remove meaningful cultural nuance and may even oversimplify the construct. Moreover, only three items per factor are few and might be statistically fragile. The authors could justify on these issues.

Factor analytic procedure is not fully appropriate:

The manuscript uses EFA and CFA on the same dataset. Although the authors mention two subsamples, it is unclear how the split was conducted and whether the EFA and CFA samples were independent. This could introduce overfitting. The authors should clarify the exact subsample sizes and whether EFA and CFA were conducted on separate datasets. Moreover, the CFA results show poor model fit for Kenya. However, the manuscript dismisses this issue too quickly and still recommends the scale for Kenya. The authors should investigate why Kenya differs, test measurement invariance more thoroughly and consider cultural heterogeneity.

Interpretation sometimes exceeds the evidence:

Several claims in the discussion are stronger than the results support. For example,

the paper claims the CSTI enables “culturally informed diagnosis and treatment.” However, the study did not test clinical outcomes and the sample was not clinical. The authors might tone down these claims.

The manuscript suggests that one item score ≥2 may be clinically significant. This is not empirically justified. Diagnostic thresholds might need a clinical validation.

Minor Concerns

The manuscript contains many grammatical errors. These appear throughout the manuscript. The paper requires professional language editing.

Examples:

• “culturally scripts of trauma sufferings”

• “the confirmatory factor analysis confirm”

• “sequalae”

• “Survivors in Namibia was perceiving their sufferance as something standing inside“-> e.g.: “Survivors in Namibia perceived their suffering as something that existed within them.“

• “exeperts“

• e.g. lines 5, 6, 23: Spaces are missing or duplicated.

Some sentences sound awkward in academic English:

• Line 11: “Those sufferings are in a new theory investigated as Cultural 12 Scripts of Trauma (CST)“ -> e.g.: “These forms of suffering are conceptualized in a new theoretical framework as Cultural Scripts of Trauma (CST).“

• Line 91: “The 20-item of Clinical Aspects of Historical Trauma Questionnaire concerns cultural patterns of men-made collective trauma, prevalent in Eastern Africa.“ -> e.g. “The 20-item Clinical Aspects of Historical Trauma Questionnaire assesses cultural patterns of man-made collective trauma prevalent in Eastern Africa.“

Methods:

• Please clarify why Rwanda was not included in the quantitative part of the study.

• Please indicate whether the questionnaires used in this study (other than the CSTI, which was developed here) have been validated for the respective cultural and linguistic contexts in which they were applied (Uganda, Tanzania, Rwanda, and Kenya).

• Please list the subscales of the Clinical Aspects of Historical Trauma Questionnaire in the methodology section. In addition, please clarify what is meant by the term “clinical aspects of historical trauma.”

• Under which ethical approvals was data collection in Tanzania and Uganda conducted? Please clarify why local ethical approval appears to have been obtained only for Kenya and Rwanda.

• Participants were included if they had “past traumatic experiences.” However, the type and severity of trauma are not clearly operationalized. The authors should clarify how trauma exposure was defined and assessed.

• Several items in the scale appear to be unrelated to trauma. For example, items such as “I am proud of my achievements” or “I believe everything is possible” might resemble general well-being or personal growth items rather than trauma-related symptoms. The conceptual rationale for including such items should be clarified.

• East Africa is culturally diverse. Combining four countries may obscure important differences in religion, conflict history, and trauma exposure. The manuscript should discuss cross-cultural variability more critically.

Results:

• Some tables are difficult to interpret, for example Table 7 (HTMT values) and Table 6 (item analysis). Clearer formatting and labeling would improve readability.

• I do not understand the age categories used in the manuscript. For example, the groups 29–44 and 41–55 appear to overlap. Please clarify the rationale for dividing the sample into these categories. I also do not understand the division into three age groups presented in Table 2. Please explain why these specific age groupings were chosen instead of reporting mean age and standard deviation.

• Relatedly, the manuscript does not report standard deviations (SDs) for several variables. Please clarify why these are not included, as SDs would help readers better understand the distribution of the data.

Discussion:

• The manuscript states: “Participants described how traumatic experiences extend beyond the individual level to affect the entire family.” This statement seems to imply that such family-level effects are specific to people in Sub-Saharan Africa. However, although these dynamics may not be explicitly reflected in diagnostic frameworks such as ICD-10 or ICD-11, this does not mean that patients in Europe do not experience similar family-level consequences of trauma. A more balanced discussion would be helpful. Are there studies from European contexts examining how PTSD-related suffering extends beyond the individual to affect the family system? From my clinical experience, such family dynamics are also highly relevant in European settings.

• The authors refer to PTSD and cPTSD in the introduction, yet this distinction is not clearly reflected in the discussion of cultural scripts. It would be helpful to clarify whether the distinction between simple and complex post-traumatic experiences plays a role within the concept of cultural scripts. This may be particularly relevant for aspects such as interpersonal relationship difficulties, which are central features of complex trauma presentations.

I recommend a Major Revision

The manuscript addresses an important topic and has strong potential. However, substantial revisions are required to:

• improve conceptual clarity

• strengthen methodological transparency

• address sampling bias

• refine statistical reporting

• correct language and presentation issues.

With these improvements, the study could make a valuable contribution to culturally informed trauma research and will, in my opinion, become a very important and widely cited paper that addresses a significant gap in global and transcultural trauma research.

Reviewer #3: I congratulate the authors to their manuscript. It advances the discussion of the PTSD concept considerably. The challenge of this manuscript is that is very complex and word limit needs to be balanced with reporting of essential information. I tried to provide some comments how to further develop this manuscript.

Abstract:

- It is confusing for a reader the fact that the pre-CSTI had 71 items is first presented in Methods and then, again presented as if a new information in Results. It might be sufficient to present the number of items in Results.

Introduction:

- Line 33 ff: Please state here the aims and if relevant hypotheses of the qualitative and quantitative studies

Methods:

- Line 43 ff: Some aspects of the qualitative study remain vague, e.g. who conducted the interviews and the analysis, the languages and settings that were involved etc. How was your approach in the analysis re the different languages, e.g. did you translate all scripts to and do the qualitative work in one of the languages?

- Line 62 – 65: Please state clearly for what reason you used social media.

- Line 62 ff: It remains vague how the quantitative data assessment took place, e.g. how participants filled the questionnaires, languages used etc.

- Line 69 ff: It would be helpful to reorder the description of your measures and to assign them to the qualitative and quantitative study.

- Line 79 ff: The description of your interview protocol guide is very brief. Please refer to the theoretical approach you followed, the way how your interview guideline was developed etc. The qualitative work needs to be better described, i.e. what were the guiding questions for the coders. The systematic how items were developed based on the qualitative data remains unclear, please elaborate on that.

- Line 74 ff: It is confusing that you present the result of the study, the final CSTI, in the methods section as if it was employed like the other questionnaires. I recommend that you describe the final CSTI at the end of the results section.

- Line 82 ff: Please provide some information on whether the employed quantitative instruments can be considered culturally appropriate and on how you made sure that validated and comparable language versions were available.

- Please find a way to clearly name the two meanings of discriminant validity in your study.

Results:

Table 1 and Table 2: The reporting of the mean of the variable age is not adequate in this table format, especially as the coefficient is in the column “Frequency”; furthermore, the SD needs to be reported, too. I recommend to restructure the table or report it in the text.

- After Line 153 the line count stopped.

- Table 3: Very important information; but it might be moved to an electronic annex.

- Interpretation of the CSTs in the different categories: E.g. you write re the third category “These accounts illustrate how, in contrast to findings from Western contexts, trauma in East African settings can deeply disrupt family functioning and contribute to a loss of hope within the family unit.” These statements seem to better fit into the discussion section. E.g. concerning the third category, it is a matter of discussion whether the disruption of families and the loss of hope are specific or universal cultural phenomena.

- EFA factor extraction: Usually, you first perform the tests that guide you on factor number and then you perform an EFA with this number of factors. Please justify your approach.

- Sample used for EFA and CFA: Did you use the same sample for both methods? Usually separate samples are recommended, e.g. to split the original sample (2/3 vs 1/3). Please justify your approach.

- Table 6: The table and its information is difficult to understand, please clarify. Especially the meaning of the coefficients should be explained (e.g. in the methods section), e.g. “Item alpha value” vs “alpha” and why in “Dimension” TS and SS are mentioned; what means “general reliability”?

- Table 7: please provide the meaning of the lines.

Discussion:

- The discussion would profit from the point of view that the categories of CSTs detected in the qualitative study also relate to the concepts behind the DSM symptom categories. The universal vs. specific cultural nature of symptoms needs to be commented on.

Typos and grammar

- Line 12 “was”

- Line 44 verb missing

- Line 63 verb missing

- Line 150: Please write “Qualitative results”

**********

what does this mean?). If published, this will include your full peer review and any attached files.

If you choose “no”, your identity will remain anonymous but your review may still be made public.

**Do you want your identity to be public for this peer review?**  For information about this choice, including consent withdrawal, please see our Privacy Policy

Reviewer #1: **Yes:**  Madison Fertig

Reviewer #2: No

Reviewer #3: No

**********

[NOTE: If reviewer comments were submitted as an attachment file, they will be attached to this email and accessible via the submission site. Please log into your account, locate the manuscript record, and check for the action link "View Attachments". If this link does not appear, there are no attachment files.]

To ensure your figures meet our technical requirements, please review our figure guidelines: https://journals.plos.org/plosone/s/figures

You may also use PLOS’s free figure tool, NAAS, to help you prepare publication quality figures: https://journals.plos.org/plosone/s/figures#loc-tools-for-figure-preparation.

NAAS will assess whether your figures meet our technical requirements by comparing each figure against our figure specifications.

Attachment Submitted filename: PLOS One- Review.docx

# Author comment: Author response to Decision Letter 1

**DOI:** 10.1371/journal.pone.0358482.r002

1 Jun 2026

Reviewers responses are included in the documents.

Attachment Submitted filename: Response to Reviewers comments.docx

# Peer review: Decision Letter 1

**DOI:** 10.1371/journal.pone.0358482.r003

**Authors:** Inga Schalinski

15 Aug 2026

Dear Dr. Mutuyimana,

Thank you for submitting your manuscript to PLOS ONE. After careful consideration, we feel that it has merit but does not fully meet PLOS ONE’s publication criteria as it currently stands. Therefore, we invite you to submit a revised version of the manuscript that addresses the points raised during the review process.

Please submit your revised manuscript by Sep 29 2026 11:59PM. If you will need more time than this to complete your revisions, please reply to this message or contact the journal office at plosone@plos.org. When you're ready to submit your revision, log on to https://www.editorialmanager.com/pone/ and select the 'Submissions Needing Revision' folder to locate your manuscript file.

- A letter that responds to each point raised by the academic editor and reviewer(s). You should upload this letter as a separate file labeled 'Response to Reviewers'.
- A marked-up copy of your manuscript that highlights changes made to the original version. You should upload this as a separate file labeled 'Revised Manuscript with Track Changes'.
- An unmarked version of your revised paper without tracked changes. You should upload this as a separate file labeled 'Manuscript'.

If you would like to make changes to your financial disclosure, please include your updated statement in your cover letter. Guidelines for resubmitting your figure files are available below the reviewer comments at the end of this letter.

If applicable, we recommend that you deposit your laboratory protocols in protocols.io to enhance the reproducibility of your results. Protocols.io assigns your protocol its own identifier (DOI) so that it can be cited independently in the future. For instructions see: https://journals.plos.org/plosone/s/submission-guidelines#loc-laboratory-protocols. Additionally, PLOS ONE offers an option for publishing peer-reviewed Lab Protocol articles, which describe protocols hosted on protocols.io. Read more information on sharing protocols at https://plos.org/protocols?utm_medium=editorial-email&utm_source=authorletters&utm_campaign=protocols.

As the corresponding author, your ORCID iD is verified in the submission system and will appear in the published article. PLOS supports the use of ORCID, and we encourage all coauthors to register for an ORCID iD and use it as well. Please encourage your coauthors to verify their ORCID iD within the submission system before final acceptance, as unverified ORCID iDs will not appear in the published article. *Only*  the individual author can complete the verification step; PLOS staff *cannot*  verify ORCID iDs on behalf of authors.

We look forward to receiving your revised manuscript.

Kind regards,

Inga Schalinski

Academic Editor

PLOS One

Journal Requirements:

If the reviewer comments include a recommendation to cite specific previously published works, please review and evaluate these publications to determine whether they are relevant and should be cited. There is no requirement to cite these works unless the editor has indicated otherwise.

Please review your reference list to ensure that it is complete and correct. If you have cited papers that have been retracted, please include the rationale for doing so in the manuscript text, or remove these references and replace them with relevant current references. Any changes to the reference list should be mentioned in the rebuttal letter that accompanies your revised manuscript. If you need to cite a retracted article, indicate the article’s retracted status in the References list and also include a citation and full reference for the retraction notice.

[Note: HTML markup is below. Please do not edit.]

Reviewers' comments:

Reviewer's Responses to Questions

**Comments to the Author**

Reviewer #2: All comments have been addressed

Reviewer #3: (No Response)

**********

2. Is the manuscript technically sound, and do the data support the conclusions?

Reviewer #2: Yes

Reviewer #3: Yes

**********

3. Has the statistical analysis been performed appropriately and rigorously?

Reviewer #2: Yes

Reviewer #3: Yes

**********

4. Have the authors made all data underlying the findings in their manuscript fully available?

The PLOS Data policy

Reviewer #2: Yes

Reviewer #3: Yes

**********

5. Is the manuscript presented in an intelligible fashion and written in standard English?

Reviewer #2: Yes

Reviewer #3: Yes

**********

Reviewer #2: I am pleased to recommend acceptance of the current manuscript for publication. The manuscript has improved substantially and is now appropriate for publication. Only a few minor editorial issues should be corrected:

• Line 30: Remove the extra “AND“.

• Line 426: The term “Western Samples” is duplicated in the sentence.

Subject to these minor corrections, I support publication of the manuscript.

Reviewer #3: Thank you for addressing my comments. The manuscript has been improved substantially.

I just have some minor comments.

Abstract:

Please mention in the Methods paragraph clearly that the quantitative survey was an online survey. EG. “In an online survey, participants completed the pre-CSTI and additional measures…”

Line 156: Please make it clearer that you used a online questionnaire by writing “The participants used a google form link to fill out the online questionnaire.”

Line 161: The instructions for subjects should be described here too.

Lines 280 ff: “These accounts illustrate how, in contrast to findings from Western contexts, trauma in East African settings can deeply disrupt family functioning and contribute to a loss of hope within the family unit.”

Again, I comment on the phrase “in contrast to findings from Western settings” which is a claim not being supported with literature in this context. I doubt that there is a contrast between findings from Western contexts and East African settings in this aspect as long as you don’t provide clear evidence. From my personal experience as expert I see every day how trauma disrupts family functioning in a western context. Maybe you want to say that this symptom dimension is not reflected in the DSM and ICD sets of symptom criteria; I would agree on that. Amyway, you should support this claim with literature, but in the results section this is not the right place for it. Better place this claim and its discussion into the discussion section and reword the respective sentence to

“These accounts illustrate how trauma in East African settings can deeply disrupt family functioning and contribute to a loss of hope within the family unit" and eventually " – what is not reflected in DSM and ICD.”

Lines 336: You may reference Table 1 in this paragraph.

Line 336 and Table 1: For me unclear why you included item No 7 as sleeping poblems are already included in the DSM and ICD lists of symptoms. Isn’t the overall aim of the CSTI to contribute additional symptoms? Please explain why you decided to include the sleeping problems into the CSTI.

Line 354, Table 2: The labeling of columns is confusing – which label refers to which column?

Line 431: “the CFA only confirm four categories”. Is this a mistake as you report 5 categories? Please explain better.

Supplemental Material 4: The instructions and explanations for subjects about to fill the CSTI should be reported here, too.

Minor comments:

Line 30: “and generally“ seems to be there by mistake as it does not make sense

Line 71: “;“

Line 212: Please introduce here the abbreviation EFA that you use below.

**********

what does this mean?). If published, this will include your full peer review and any attached files.

If you choose “no”, your identity will remain anonymous but your review may still be made public.

**Do you want your identity to be public for this peer review?**  For information about this choice, including consent withdrawal, please see our Privacy Policy

Reviewer #2: No

Reviewer #3: No

**********

[NOTE: If reviewer comments were submitted as an attachment file, they will be attached to this email and accessible via the submission site. Please log into your account, locate the manuscript record, and check for the action link "View Attachments". If this link does not appear, there are no attachment files.]

To ensure your figures meet our technical requirements, please review our figure guidelines: https://journals.plos.org/plosone/s/figures

You may also use PLOS’s free figure tool, NAAS, to help you prepare publication quality figures: https://journals.plos.org/plosone/s/figures#loc-tools-for-figure-preparation.

NAAS will assess whether your figures meet our technical requirements by comparing each figure against our figure specifications.

# Author comment: Author response to Decision Letter 2

**DOI:** 10.1371/journal.pone.0358482.r004

31 Aug 2026

The revision are included in cover letter.

# Editor report: Decision Letter 2

**DOI:** 10.1371/journal.pone.0358482.r005

**Authors:** Inga Schalinski

2 Sep 2026

Eastern African Expressions of Suffering: a new Cultural Scripts of Trauma Inventory

PONE-D-25-53823R2

Dear Dr. Mutuyimana,

We’re pleased to inform you that your manuscript has been judged scientifically suitable for publication and will be formally accepted for publication once it meets all outstanding technical requirements.

Within one week, you’ll receive an e-mail detailing the required amendments. When these have been addressed, you’ll receive a formal acceptance letter and your manuscript will be scheduled for publication.

An invoice will be generated when your article is formally accepted. Please note, if your institution has a publishing partnership with PLOS and your article meets the relevant criteria, all or part of your publication costs will be covered. Please make sure your user information is up-to-date by logging into Editorial Manager at Editorial Manager® and clicking the ‘Update My Information' link at the top of the page. For questions related to billing, please contact billing support.

If your institution or institutions have a press office, please notify them about your upcoming paper to help maximize its impact. If they’ll be preparing press materials, please inform our press team as soon as possible -- no later than 48 hours after receiving the formal acceptance. Your manuscript will remain under strict press embargo until 2 pm Eastern Time on the date of publication. For more information, please contact onepress@plos.org.

Kind regards,

Inga Schalinski

Academic Editor

PLOS One

Additional Editor Comments (optional):

Reviewers' comments:

# Editor report: Acceptance letter

**DOI:** 10.1371/journal.pone.0358482.r006

**Authors:** Inga Schalinski

PONE-D-25-53823R2

PLOS One

Dear Dr. Mutuyimana,

I'm pleased to inform you that your manuscript has been deemed suitable for publication in PLOS One. Congratulations! Your manuscript is now being handed over to our production team.

At this stage, our production department will prepare your paper for publication. This includes ensuring the following:

* All references, tables, and figures are properly cited

* All relevant supporting information is included in the manuscript submission,

* There are no issues that prevent the paper from being properly typeset

You will receive further instructions from the production team, including instructions on how to review your proof when it is ready. Please keep in mind that we are working through a large volume of accepted articles, so please give us a few days to review your paper and let you know the next and final steps.

Lastly, if your institution or institutions have a press office, please let them know about your upcoming paper now to help maximize its impact. If they'll be preparing press materials, please inform our press team within the next 48 hours. Your manuscript will remain under strict press embargo until 2 pm Eastern Time on the date of publication. For more information, please contact onepress@plos.org.

You will receive an invoice from PLOS for your publication fee after your manuscript has reached the completed accept phase. If you receive an email requesting payment before acceptance or for any other service, this may be a phishing scheme. Learn how to identify phishing emails and protect your accounts at https://explore.plos.org/phishing.

If we can help with anything else, please email us at customercare@plos.org.

Thank you for submitting your work to PLOS One and supporting open access.

Kind regards,

PLOS One Editorial Office Staff

on behalf of

Dr. Inga Schalinski

Academic Editor

PLOS One
